# 16S rRNA Gene-Based Metagenomic Analysis of Rhizosphere Soil Bacteria in Arkansas Rice Crop Fields

**DOI:** 10.3390/agronomy12010222

**Published:** 2022-01-17

**Authors:** Cássia Oliveira, Ehsan Shakiba, Dustin North, Madison McGraw, Ethan Ballard, Marissa Barrett-D’Amico, Galina Glazko, Yasir Rahmatallah

**Affiliations:** 1 Science Division, Lyon College, Batesville, AR 72501, USA; 2 Rice Breeding and Genetics, Rice Research & Extension Center, University of Arkansas, Stuttgart, AR 72160, USA; 3 College of Medicine, University of Arkansas for Medical Sciences, Little Rock, AR 72205, USA; 4 School of Medicine, Duke University, Durham, NC 27710, USA; 5 Department of Biomedical Informatics, University of Arkansas for Medical Sciences, Little Rock, AR 72205, USA

**Keywords:** crop rotation, Illumina MiSeq sequencing, microbial soil diversity, rice, tillage, 16S rRNA gene

## Abstract

The rhizomicrobiome is composed of microbes that live in association with plant roots. From nutrient cycling to carbon sequestration, soil microorganisms have provided a solid base for natural and agricultural ecosystems to function. The relationship between plant roots and soil microorganisms is especially relevant in food staples such as rice (*Oryza sativa* L.), as the various properties of these microbes can influence crop yield and plant health, thereby affecting a major portion of the food supply for an ever-growing world population. In this study, we used 16S rRNA gene-based metagenomic analysis to investigate the impact of crop rotation and soil cultivation methods (no-till or tillage) on rhizosphere bacterial diversity and composition in eight crop fields in Arkansas. Illumina MiSeq sequencing revealed 56 Phyla, with four major Phyla: Proteobacteria, Acidobacteria, Actinobacteria, and Bacteroidetes. Soil microbial communities in the samples studied were phylogenetically diverse but with a stable community structure. Crop rotation and tillage did not significantly affect bacterial diversity.

## 1. Introduction

Soil microbial communities are the drivers of many ecological functions [1,2], can affect plant species diversity and productivity [3], and are a crucial component of agricultural systems. The soil microbiota, composed of archaea, bacteria, fungi, and protists, has a vital role in natural and agroecosystems, mainly by regulating carbon and nitrogen cycling and fostering plant growth and productivity. Despite the pivotal roles microbes play in sustaining life on Earth [4], our knowledge of their diversity, ecology, and interactions is limited. Over 99% of microbes are not amenable for cultivation in a laboratory setting [5]. Nowadays, such limitation can be circumvented with metagenomics—a technique that allows the identification of microbes directly from environmental samples. In this way, multiple genomes can be analyzed simultaneously by extracting the DNA from all the microorganisms found in a sample of interest [6]. In this study, we investigated the impact of crop type (no rotation (rice monoculture) and rotation (with or without rice in the rotation)) and soil cultivation methods (no-till or tillage) on rhizosphere bacterial diversity and composition in eight crop fields in Arkansas using 16S rRNA gene-based metagenomic analysis.

### 1.1 Rice as a Major Crop in Arkansas and the World

Rice is considered one of the most vital grains globally due to its worldwide agricultural importance as a food staple [7]. In 2018, the United States ranked as the twelfth major rice producer and fifth major rice exporter in the world [8]. Rice is grown in seven US states, Arkansas, California, Florida, Louisiana, Mississippi, Missouri, and Texas. Arkansas is the major rice producer in the country, with approximately 50% of total US rice production [9].

The conventional method of rice irrigation in Arkansas is flooding. Seeds are planted in drilled dry seeded rows; levees are constructed around the field and flooded when plants reach the V4 stage. Conventional tillage and no-tillage methods are employed in rice fields [9]. Fertilizer utilization is also a common practice in rice production, with nitrogen as the most commonly used fertilizer [10,11]. Crop rotation, especially with legumes, is also widely used as it promotes higher crop productivity and benefits the soil’s chemical, physical, and biological properties [12–15]. Several studies have focused on the biological properties of soils, including interactions between plants and their associated root microbial communities—the rhizosphere. In the past decades, scientists have uncovered a number of microbial pathogens and microbial symbionts that can affect plant growth and health, with the interaction between rhizobia and mycorrhizal fungi as a classic example [16,17].

### 1.2 Soil Microbiome

The co-evolution between plants and associated microbial communities dates back to the evolution of terrestrial plants, 450 million years ago. Ultimately, it led to the evolution of mutualistic, commensalistic, and pathogenic relationships [18]. Soil is a functional and vital living system, and soil health is required to sustain agricultural productivity and promote plant and animal welfare while maintaining or enhancing water and air quality. Soil microorganisms are indispensable to the living soil and serve as soil health indicators [19]. In the past few years, major advances in molecular analysis have opened the way towards a deeper understanding of plant–microbe interactions, ranging from composition to structure to function. We can use such knowledge to solve modern-day agricultural problems. For instance, disease-suppressive soils are a well-known phenomenon where microorganisms in the rhizosphere (soil close to the root surface) assist plants in fighting off soil-borne pathogens via mostly as-yet unknown mechanisms [16]. *Rhizoctonia solani* is a root-pathogenic fungus that affects many economically important crops such as beet, potato, and rice [20]. Mendes et al. [21] used a PhyloChip-based metagenomics approach and culture-dependent functional analysis to identify the bacterial taxa and genes involved in suppressing *R. solani*. The 16S rRNA sequencing of samples taken from rice root compartments has revealed distinct microbiomes that provide beneficial interaction with plant roots [22]. Another study used RNA-sequencing of samples taken from rice roots that were colonized with nitrogen-fixing bacteria and showed a relationship to plant growth promotion, as well as differential gene expression related to flavonoid biosynthesis pathways, nitrate transporters, defense pathways, and hormone signaling [23]. Metagenomic approaches have also been employed to identify microbial genes present in the rice microbiome and their association with plant hormone metabolism, pathogenic resistance, methanol oxidation, and nitrogen fixation [24,25].

## 2. Materials and Methods

### 2.1 Soil Sampling and Processing

Soil samples were collected from eight locations in Arkansas (Table 1) and, except for location F (Fayetteville), all other collection sites were located in the Arkansas rice-growing region. Three locations had no crop rotation, i.e., only rice was planted as a crop. These locations were named B, C, and D and belonged to Isbell Rice Farms. On this private commercial rice farm, monocrop rice has been the only agricultural practice for the past 48, 57, and 50 consecutive years, respectively. The next three locations had crop rotations between rice and soybean and were called locations E, G, and H. In location E, found at the Arkansas Agricultural Experiment Station, Rice Research and Extension Center (RREC), rice and soybean are alternated every year (1 year rice: 1 year soybean). Location G, known as North, has a rotation of 2 years soybean: 1 year rice. Location H, in Stratton, has a rotation of 3 years soybean: 1 year rice. Finally, two locations had no rice in their rotations. Location A (RREC), has a rotation of 2 years corn: 1 year cotton, and location F in Fayetteville has a rotation of 1 year corn: 1 year soybean (Table 1). In all five locations with crop rotation, the practices had been performed for at least 10 years. The soil cultivation method employed in the eight study sites was either no-till, where the soil hardpan layer remained intact, or tillage, where the hardpan layer was broken by mechanical means using a plow machine. In addition, field management activities, such as fertilizer application, were implemented according to the standard rice growth recommendations for Arkansas [9].

All soil samples were collected in mid-November 2017, after harvesting the crops, but before the cultivation of the soil. The number of soil samples collected ranged from 4–8, depending on the field size. Each sample was collected in the root zone area (rhizosphere) using a shovel, within ca. 10.5 cm depth for row crop fields, i.e., rice, soybean, and corn. Samples were stored in a cold room at 4 °C until DNA was extracted (see below). Soil physical and chemical composition analyses were performed at the University of Arkansas soil test laboratory, Keiser, AR.

### 2.2. DNA Extraction and DNA Sequence Analysis

Total DNA was extracted from freshly collected soil samples (within three months after collection) using PowerLyzer^®^ PowerSoil^®^ DNA isolation kit (MO BIO Laboratories, Inc., Carlsbad, CA, USA) according to the manufacturer’s instructions. DNA was quantified using a Qubit^™^ 3.0 Fluorometer (Life Technology Ltd., Paisley, UK). The presence of DNA was also verified by gel electrophoresing 10 μL of total DNA on 2% agarose using SYBR^®^ safe DNA gel stain (Invitrogen, Groningen, the Netherlands). The 260/280 ratio was measured using a Biophotometer (Eppendorf, Hamburg, Germany). DNA samples were stored at −20 °C until DNA sequencing was performed.

Two samples from each of the eight study locations (except for location G, where three samples were used instead of two) were sequenced at the University of Arkansas for Medical Sciences (UAMS) Sequencing Core Facility. V3 and V4 regions of the bacterial 16s rRNA gene were amplified using primers containing Illumina adapters following Illumina’s 16S Metagenomics Protocol (Part # 15,044,223 Rev. B). Briefly, the Kapa Library Amplification Kit (Kapa Biosystems, Wilmington, MA, USA) was used for PCR, and products were cleaned using Beckman Coulter Agencourt AMPure XP Beads (Beckman-Coulter, Pasadena, CA, USA) according to the 16S Metagenomics protocol. We used universal primers reported by Klindworth et al. [26]. Forward and reverse primer sequences were, 5*′*-TCGTCGGCAGCGTCAGATGTGTATAAGAGACAGCCTACGGGNGGCWGCAG-3*′* and 5*′*-GTCTCGTGGGCTCGGAGATGTGTATAAGAGACAGGACTACHVGGGTATCTAATCC-3*′*, respectively, and created a single amplicon of approximately 460 bp. Concentrations were adjusted to 4 uM and prepared for loading on the Illumina MiSeq according to Illumina’s 16S Metagenomics Protocol (Part # 15,044,223 Rev. B). Samples were pooled, denatured, and loaded on the Illumina MiSeq at 8 pM and sequenced paired-end (2 300) using a MiSeq ^®^ Reagent Kit v3 (600 cycles) (Illumina, Inc., San Diego, CA, USA).

### 2.3. Bioinformatics Analysis

The 16S rRNA sequencing data is available at the NCBI Sequence Read Archive (SRA) (http://www.ncbi.nlm.nih.gov/sra) (accessed on 15 December 2021) under accession number PRJNA782652. Raw sequence data were processed in multiple steps using the Quantitative Insights into Microbial Ecology (QIIME) pipeline [27]. First, the 300 bp paired-end reads were joined using the fastq-join method with a minimum allowed overlap of 120 bp and 15% maximum allowed difference within the overlap region. Second, reads with more than three consecutive base calls having Phred score <20 were truncated, reads with any ambiguous base call were discarded, and reads from different samples were tagged with sample identifiers and merged into a single FASTA file. Third, sequence reads were aligned against the core reference alignments of the GreenGenes database (GG13_5; https://greengenes.lbl.gov) (accessed on 15 December 2021) [28] using pyNAST [29], and operational taxonomic units (OTUs) were identified at the 97% DNA similarity level using UCLUST [30]. The counts of each OTU were normalized by the total number of aligned reads per sample. Sequences that failed the closed-reference alignment to the GreenGenes database were aligned de novo, and OTUs with <2 aligned sequences were discarded.

Alpha and beta diversity metrics were calculated in QIIME to estimate bacterial community composition. Alpha diversity (within-sample diversity) was measured using the observed number of OTUs, phylogenetic distance (PD), and Chao1 [31,32] and visualized as rarefaction plots. Beta diversity (between-sample diversity) was estimated using unweighted Unifrac pairwise sample dissimilarity in OTU abundance profiles among samples [33,34]. Principal component analysis (PCA) and hierarchical clustering were used to visualize the results.

We calculated the correlation between bacterial community structure and soil properties (Table 2) using non-metric multidimensional scaling (NMDS) as implemented in the vegan package in R [35]. Distance matrices were constructed using Bray–Curtis and Euclidean distances. ANOVA was performed to identify environmental factors (predictors or independent variables) that influence the diversity index (response or dependent variable). Type I (sequential) ANOVA returned drastically different results when the order of the predictors changed; hence, we used a Type II ANOVA method that was independent of the order of predictors in the model. We used the Shannon diversity index, a measure of within-sample variation, as the response variable and P, K, Ca, Mg, SO4-S, Zn, Fe, Mn, Cu, B, pH, rotation, and till as the predictors. Predictors were ordered by their absolute pairwise Pearson’s correlation coefficient with the Shannon diversity index.

## 3. Results and Discussion

The number of merged reads aligned successfully to each sample varied among the eight locations studied. Sample 3 (location B, rice monoculture) had the lowest number (372,886), whereas sample 7 (location D, rice monoculture) had the highest number (794,318). The percentage of reads passing quality filtering was 95% or higher for all 17 samples (Table 3). Given the rarity of Archaea in our dataset (0.0–0.1%), we decided to omit Archaea and focus on the members of the bacteria domain. Among 221,105 detected OTUs, we considered 170,853 OTUs associated with bacteria. While close-reference OTU-picking detected 14,474 OTUs, 156,379 OTUs were picked de novo. Detected OTUs were assigned to 56 Phyla (100% of OTUs), 164 classes (98.4% of OTUs), 261 orders (87.9% of OTUs), 314 families (56.8% of OTUs), 522 genera (23.4% of OTUs), and 190 species (1% of OTUs).

**Figure 1.**
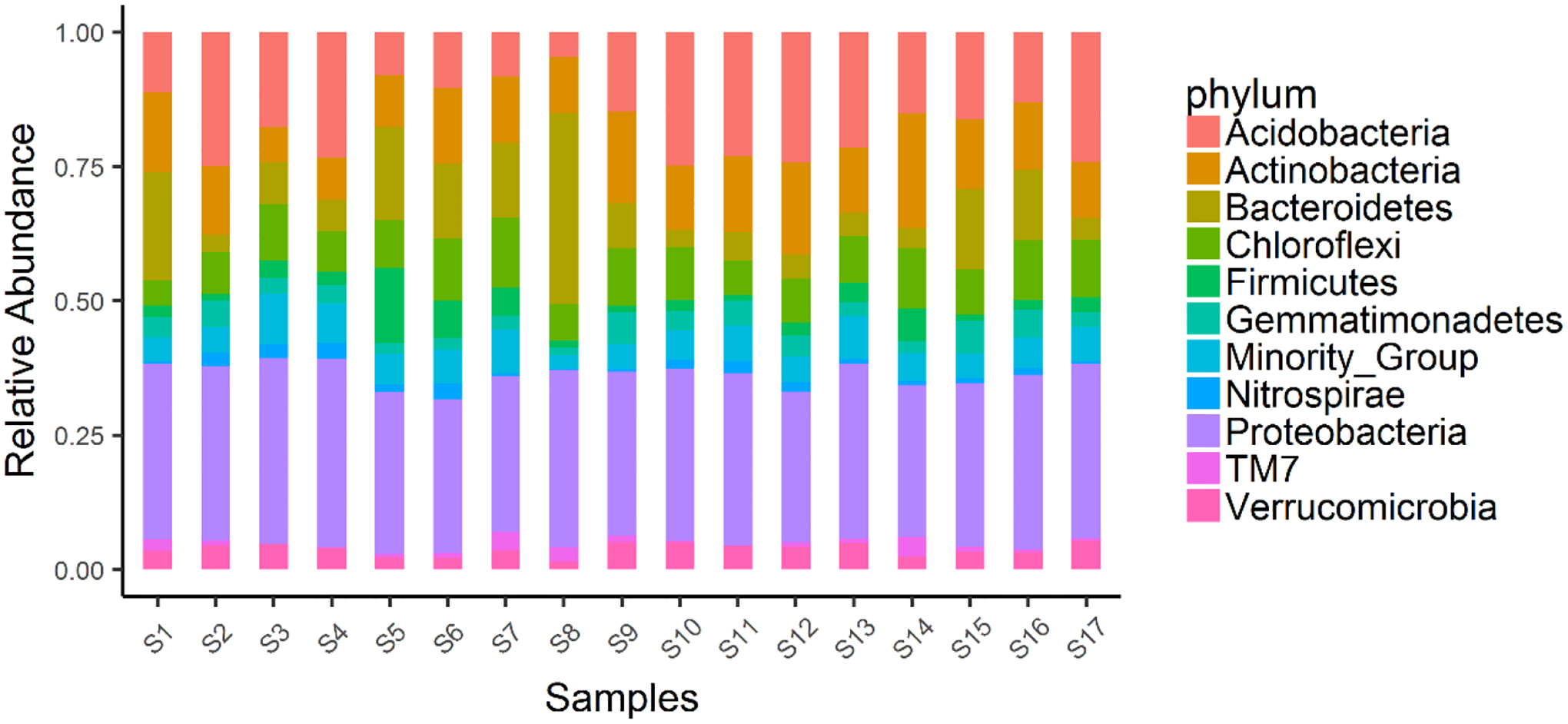
Barplot illustrating bacterial diversity at the Phylum level for rice/crop rhizosphere soil. Only the top ten Phyla by average relative abundance across samples are shown, and all other Phyla were merged into one group called the minority group. Relative abundance of each bacterial Phylum refers to the proportion of reads aligned to OTUs associated with the phylum in each sample. See Table 1 for soil sample location description.

Out of the 56 Phyla identified, ten were considered major Phyla by average relative abundance across samples among the eight locations studied. The ten most prominent Phyla were Proteobacteria (average: 30.7%; range: 27.4–34.4%), followed by Acidobacteria (16.4%; 4.6–24.40%), Actinobacteria (12.5%; 6.5–20.8%), Bacteroidetes (10.3%; 3.1–35.10%), Chloroflexi (9%; 4.6–12.8%), Verrucomicrobia (3.7%; 1.5–5.20%), Gemmatimonadetes (3.5%; 1.3–6%), Firmicutes (3.3%; 1–13%), Nitrospirae (1.4%; 0.20–2.8%), and TM7 (1.2%; 0.1–3.60%). Figure 1 shows the relative abundance of the top ten bacterial Phyla in all 17 samples. The other Phyla were merged together into one group, called minority group, as indicated in the legend. All ten major Phyla have been described as dominant taxa in soil studies [36,37]. Planctomycetes was the only Phylum with low representation in our samples (0.2–1.2%) but commonly listed as a major group in soil studies.

At the class level, alpha-, beta-, and delta-Proteobacteria were the top three classes across the 17 samples with a total percentage of reads of 10.9%, 9.30%, and 6.8%, respectively. Gamma-proteobacteria ranked as number eight. Other top classes included Actinobacteria and Thermoleophilia (Phylum Actinobacteria); Solibacteres; Acidobacteriia; Chloracidobacteria; Acidobacteria-6 (Phylum Acidobacteria); Anaerolineae (Phylum Chloroflexi); Saprospirae; Bacteroidia; Flavobacteriia (Phylum Bacteroidetes); Pedosphaerae (Phylum Verrucomicrobia); and Gemmatimonadetes (Phylum Gemmatimonadetes) (Supplemental Table S1). Differences among locations included the presence of Bacteroidia as the second most common class in location C (rice monoculture). In contrast, Flavobacteriia was the most prevalent class in location D (rice monoculture).

**Figure 2.**
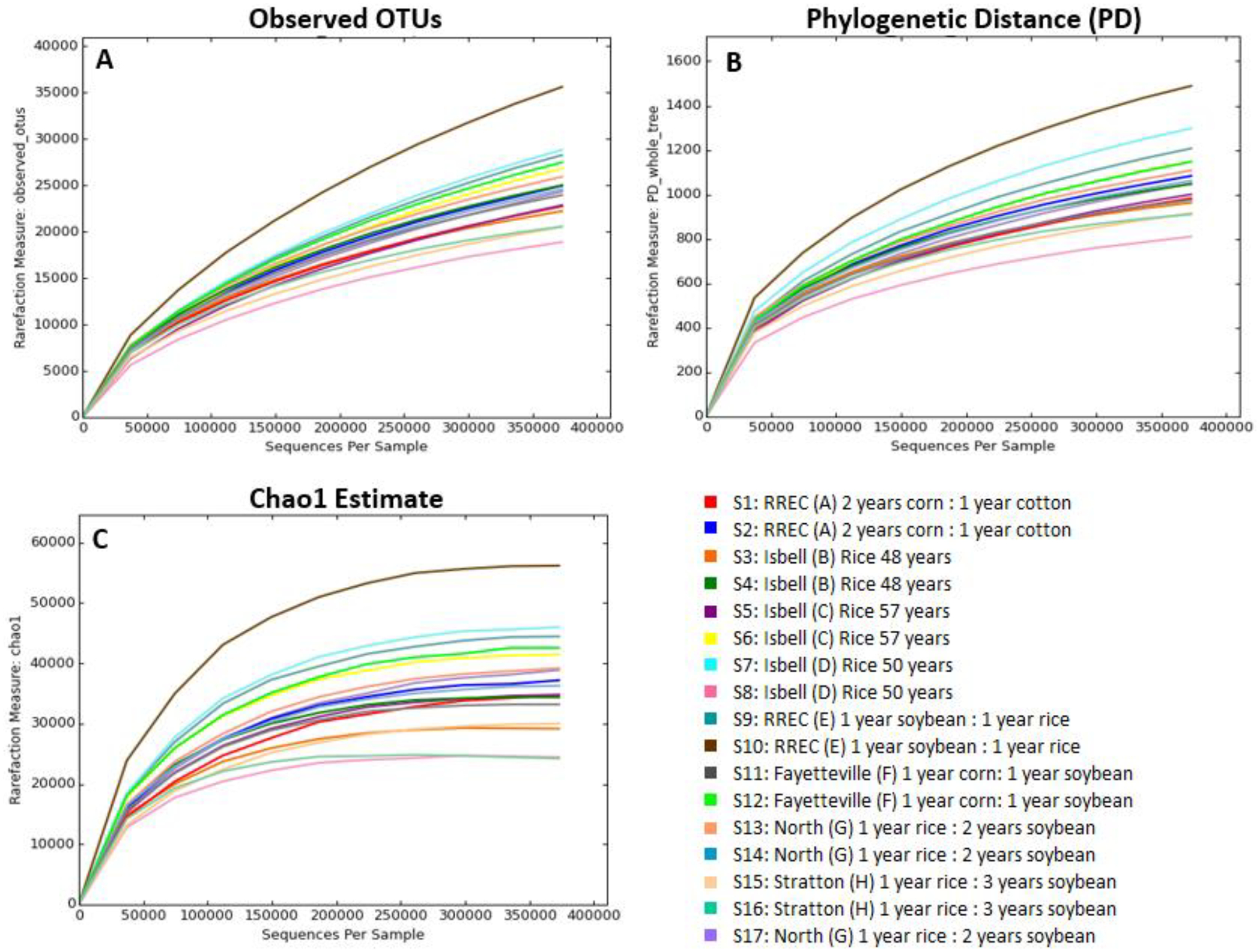
(**A**–**C**). Alpha diversity analysis of the rhizosphere microbiome in eight agricultural fields in Arkansas. (**A**–**C**): Rarefaction plots showing the observed number of OTUs (Operational Taxonomic Units), phylogenetic distance (PD), and Chao1 estimate, respectively, for the 17 soil samples analyzed. S1–S17 = Metagenomic sample ID followed by field name, location, and type of culture: rice monoculture or crop rotation.

The rarefaction plot for the observed number of OTUs showed that sample 10 (location E), had the highest number of OTUs. In this location, rice is rotated every year with soybean, which can help explain the high diversity of bacteria. We discuss the importance of crop rotation later on in the paper. In contrast, sample 8 (location D, rice monoculture) had the lowest number of OTUs (Figure 2A). While OTU count allows pure estimates of community richness, phylogenetic distance (PD) provides additional information as it accounts for the degree of phylogenetic divergence between sequences within each sample [31]. The rarefaction plot based on the PD (Figure 2B) produced similar results to the OUT count, even though biodiversity was not fully captured in our samples (rarefaction curves were not parallel to the X-axis). Similar results have been observed in other studies of soil bacterial diversity [38]. Chao1 metric, which estimates the number (richness) and distribution (evenness) of taxa expected within a single sample or environment [32], revealed that sample 10 (location E, crop rotation) had the highest diversity. On the other hand, sample 8 (location D, rice monoculture) had the lowest diversity and was dominated by members of the Phylum Bacteroidetes (35.10%), class Flavobacteriia (19.75%) (Figure 2C).

**Figure 3.**
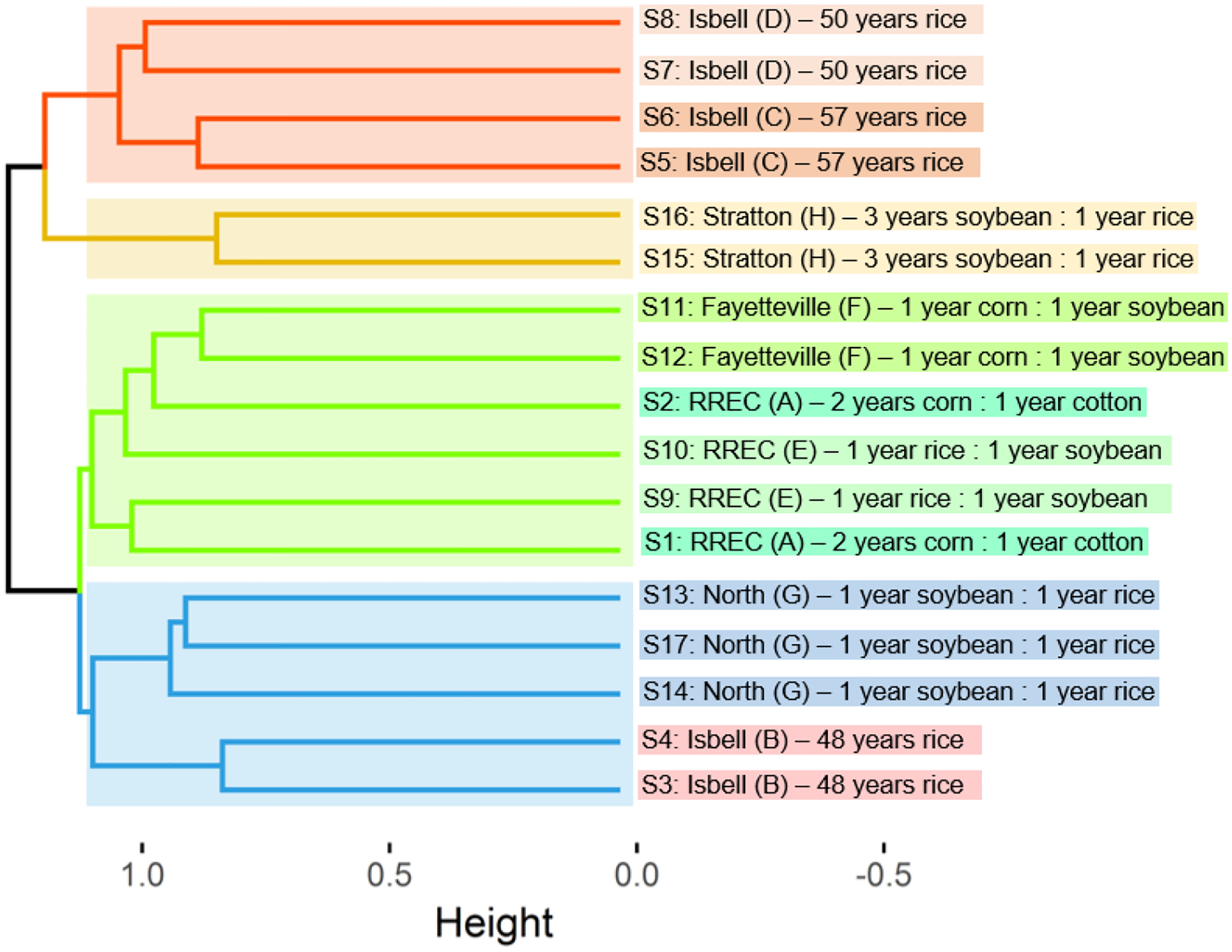
Dendrogram plot showing the similarity of the rhizosphere microbial communities in eight agricultural fields in Arkansas. S1–S17 = Metagenomics sample ID followed by field name, location (A–H), and type of culture: rice monoculture or crop rotation.

**Figure 4.**
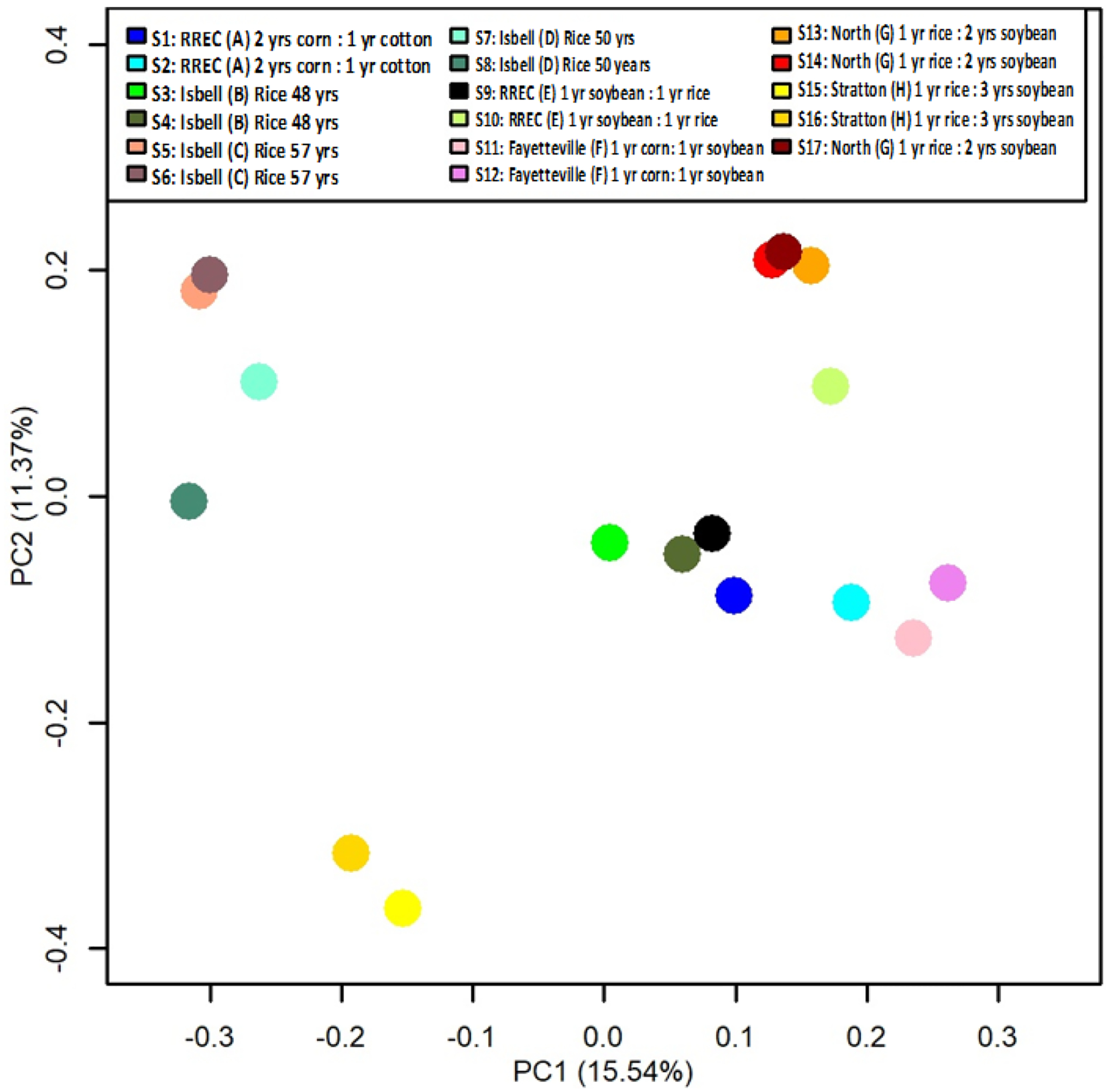
Principle component analysis (PCA) using the unweighted Unifrac metric colored by field site. S1–S17 = Metagenomics sample ID followed by field name, location (A–H), and type of culture: rice monoculture or crop rotation.

Hierarchical clustering based on unweighted Unifrac pairwise sample dissimilarity produced a tree with two clades further divided into two smaller clades (Figure 3). The dendrogram showed that replicated samples from each of the eight locations shared more similarities in bacterial community structure as they clustered together. The one exception was location B (rice monoculture), which clustered with the location G samples (crop rotation) rather than the other two locations with rice monoculture. Overall, the location where the soil samples were collected was predictive of bacterial composition, but the separation between rice monoculture and crop rotation was unclear. Principal component analysis (PCA) of the unweighted UniFrac matrix revealed a similar conclusion. In other words, samples from the same location were more similar in bacterial composition and could be subdivided into two groups: rice monoculture (locations B, C, and D) and crop rotation (locations A, E, F, G, and H) (Figure 4).

Several studies have shown that soil microbial community composition is dynamic and can be influenced by many factors, including soil type [39], pH [40], salinity [41], temperature [4], water content [42], and nutrient availability [3]. Furthermore, similar microbial composition is often observed in closely resembling environments. For example, microbial communities in deserts are taxonomically, phylogenetically, and functionally closer than in non-desert soils [4]. Forests and agricultural fields have distinct microbial compositions [43] but are more similar than subterranean environments [44].

Crop rotation and tillage, two common practices in agriculture, can also affect soil microbial makeup. Crop rotation has been historically used in agriculture because of its benefits to soil’s abiotic and biotic characteristics [12–15]. However, some studies have shown microbial diversity did not change and decreased under crop rotation [45,46]. A meta-analysis to assess the impact of crop rotation on soil microbial diversity revealed that the aboveground vegetation (presence or absence of rotation with legumes) did not affect the belowground microbial diversity or richness consistently [47]. Tillage can lead to soil disturbance by increasing soil erosion and nutrient runoff. In contrast, no-till increases soil compaction. Both procedures, till and no-till, can negatively affect crop production by interfering with plant growth due to nutrient deficiency or poor root development [48,49]. Furthermore, soil conditions can affect the soil microorganisms’ habitat, lowering microbial diversity and abundance and negatively affecting crop quality and yield [50].

**Figure 5.**
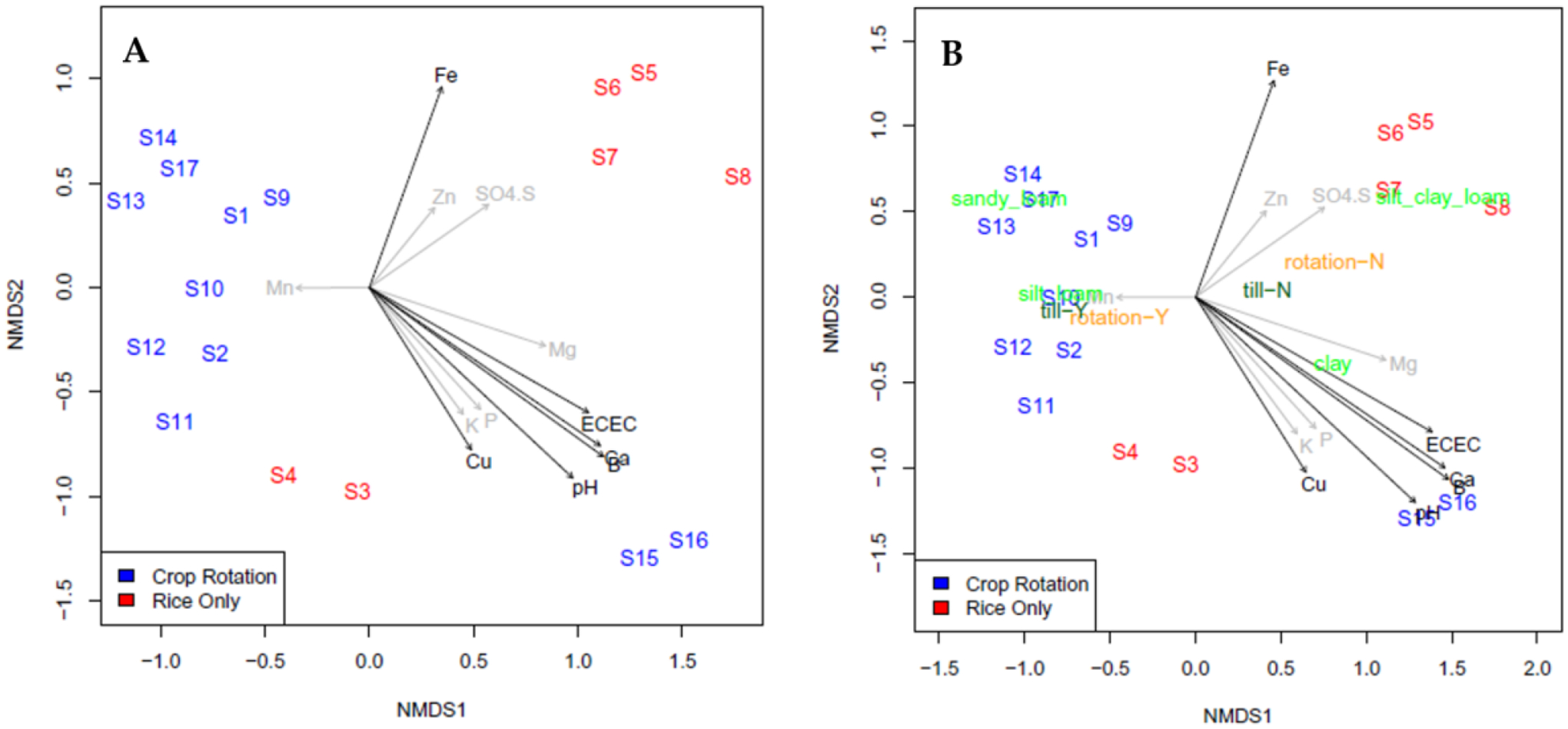
(**A**,**B**). Non-metric multidimensional scaling (NMDS) was applied to OTU abundances and fitted the environmental factors: P, K, Ca, Mg, SO4-S, Zn, Fe, Mn, Cu, B, pH, ECEC in (**A**); and rotation, texture, and till were also added to these in (**B**). Samples from fields/locations planted with only rice (no crop rotation) are colored in red, whereas samples from fields/locations were crop rotation was practiced (including rice and no rice) are colored in blue. Grey arrows = non-significant environmental factors; black arrows = significant factors (*p* < 0.05).

In our analysis, Pearson correlation coefficients between different factors and the Shannon diversity index showed that crop rotation did not significantly affect bacterial diversity (*p* = 0.2069; Table 4), whereas tillage was marginally significant (*p* = 0.0669; Table 4). All of the mineral nutrients we analyzed significantly affected the bacterial profile, except for magnesium and potassium. pH was only marginally significant (Table 4). Finally, NMDS analysis using OTU abundances and environmental factors (see Table 4) revealed that samples from locations C and D (rice monoculture fields), with no crop rotation and no-till, occupied the top-right quadrant of the NMDS scatter plot. Samples from location H with crop rotation and no-till occupied the bottom-right quadrant of the NMDS scatter plot. The rest of the samples occupied the left side (Figure 5A,B). It was also noticeable that all samples from soil with clay texture (locations B, C, D, and H) could be separated from all other samples by a straight line (see Figure 4 or Figure 5), implying that soil texture plays a major role in sample diversity at the OTU level. With the exception of samples from location G, the same separation was possible between samples with till and no-till. Unfortunately, most samples with no-till came from clay soil, and the two factors (till and clay texture) may play a confounding effect that is hard to separate with statistical models using the current data.

In a study comparing soil microbial communities in crop fields and forests, Jangid et al. [43] observed that microbial composition and abundance were primarily determined by land-use history rather than vegetation and soil properties. Moreover, the authors observed that crop fields displayed lower abundance and composition of many bacterial Phyla compared to forests. Although we did not include soil from non-agricultural fields in our analysis, our results are in agreement with Jangid et al. [43]. Namely, the type of vegetation aboveground (rice monoculture or crop rotation) did not significantly influence the microbial makeup belowground. Regarding till and no-till, the practice was not a significant contributor to bacterial diversity either. Although till and non-till practices can negatively affect crop production [49], the high level of microbial biodiversity in our study sites suggests that compensation mechanisms are in place in each location to maintain high soil productivity. Further studies are required to confirm this assertion.

## 4. Conclusions

Present-day agriculture faces a tremendous challenge by the increased global demand for food, as the human population is projected to reach nine billion people by 2050 [51]. The success of tomorrow’s agriculture will rely on improving current practices and exploring new alternatives. Our study assessed the impact of farming practices, i.e., crop rotation and tillage, on the rhizosphere bacterial diversity and abundance of rice fields in Arkansas using 16S rRNA gene-based metagenomic analysis. Our results revealed differences in bacterial composition and abundance in the eight study sites, but crop rotation and tillage were not significant contributors to the variation observed. Knowledge of the rhizosphere microbiome can lead to the creation of microbial-based biofertilizers to increase plant performance and decrease the use of traditional fertilizers and land [52]. Ultimately, such knowledge can have a significant economic and environmental impact.

## Supplementary Material

Supplementary Material

**Supplementary Materials:** The following supporting information can be downloaded at: https://www.mdpi.com/article/10.3390/agronomy12010222/s1, Table S1: Class level diversity for the eight locations analyzed (17 samples). See Table 1 for location details.

## Figures and Tables

**Table 1.** GPS coordinate locations for the eight study fields where soil was collected. Crop rotation scheme adopted shown in the last three columns. In locations with crop rotation (A, E, F, G, and H), the practice has been performed for at least 10 years.

Location	Field	Coordinates	Last Crop	Current Crop (Nov. 2017)	Rotation
A	RREC	34°27′44″ N 91°24′08″ W	Corn	Cotton	2 years corn: 1 year cotton
B	Isbell	34°34′56″ N 91°45′47″ W	Rice	Rice	Rice 48 years
C	Isbell	34°35′46″ N 91°45′28″ W	Rice	Rice	Rice 57 years
D	Isbell	34°37′32″ N 91°45′12″ W	Rice	Rice	Rice 50 years
E	RREC	34°27′48″ N 91°25′10″ W	soybean	Rice	1 year soybean: 1 year rice
F	Fayetteville	36°05′56″ N 94°10′23″ W	Corn	Soybean	1 year corn: 1 year soybean
G	North	34°17′19″ N 91°34′32″ W	Rice	Soybean	1 year rice: 2 years soybean
H	Stratton	34°29′42″ N 91°34′42″ W	Rice	Soybean	1 year rice: 3 years soybean

**Table 2.** Physical and chemical properties of soil in eight crop study fields in Arkansas. Two soil samples were analyzed for each of the eight locations, with the exception of location G (North), where three soil samples were analyzed.

Location (Field)	P	K	Ca	Mg	SO_4_-S	Zn	Fe	Mn	Cu	B	Soil pH (1:2 Soil- Water)	Soil ECEC (cmolc/kg)	Estimated Soil Texture	Crop Rotation Yes/No	Till Yes/No
A (RREC)		109	1033	69	13	3.0	141	190	0.8	0.3	6.3	9	Silt loam	Yes 2 years corn: 1 year cotton	Yes
	19	131	1097	75	11	6.2	136	223	1.5	0.3	6.3	9			
B (Isbell)	12	279	3289	850	65	3.2	263	53	7.1	0.7	6.4	29	Clay	No Rice 48 years	No
	20	376	2715	631	84	4.3	337	63	4.0	0.8	5.8	25			
C (Isbell)	11	237	3753	728	29	3.6	369	116	2.0	0.7	6.8	29	Clay	No Rice 57 years	No
	10	196	3142	674	26	3.0	406	166	1.9	0.7	6.9	25			
D(Isbell)	49	161	1700	266	119	7.1	434	28	3.2	0.7	6.5	15	Silt loam—Silty clay	No Rice 50 years	No
	57	158	1869	276	259	6.1	388	35	3.3	0.7	6.5	16			
E (RREC)	26	71	600	89	12	5.5	382	189	1.0	0.4	5.7	8	Silt loam	Yes 1 year soybean: 1 year rice	Yes
	16	69	649	101	9	6.7	225	202	1.4	0.3	5.9	7			
F (Fayetteville)	21	61	659	44	7	1.2	61	149	1.3	0.1	6.6	6	Silt loam	Yes 1 year corn: 1 year soybean	Yes
	30	80	618	42	7	1.4	74	165	1.4	0.2	6.3	6			
G (North)	44	272	367	72	21	3.7	262	12	1.1	0.2	5.3	8	Sandy loam Silt Loam	Yes 1 year rice: 2 years soybean	No
	67	220	483	101	18	4.7	343	20	1.0	0.3	5.2	9			
	25	174	534	85	148	4.3	523	11	0.3	0.4	4.6	10			
H (Stratton)	81	236	3619	338	19	3.5	174	67	2.1	1.1	8	24	Clay	Yes 1 year rice: 3 years soybean	No
	101	375	4895	390	33	4.9	201	66	3.1	1.5	8.1	31			

P = phosphorus, K = potassium, Ca = calcium, Mg = magnesium, SO4-S = sulfate-sulfur, Zn = zinc, Fe = Iron, Mn = manganese, Cu = copper, and B = boron, ECEC = effective cation exchange capacity. Two types of soil cultivation methods were employed: tillage or no-till, which led to the absence or presence of soil hardpan layer, respectively.

**Table 3.** 16S rRNA metagenomic sequencing statistics.

Location	Field	Metagenomics Sample ID	Total Reads	Reads Passing Quality Filtering	% Reads Passing Quality Filtering	Aligned Merged Reads per Sample
A	RREC	S1	936,364	899,156	96.0	749,518
		S2	771,282	743,354	96.4	617,111
B	Isbell	S3	483,099	464,663	96.2	372,886
		S4	623,931	594,092	95.2	460,853
C	Isbell	S5	892,264	860,061	96.4	725,334
		S6	909,293	875,900	96.3	713,702
D	Isbell	S7	1,028,520	980,191	95.3	794,318
		S8	597,720	576,338	96.4	478,296
E	RREC	S9	906,681	873,317	96.3	730,662
		S10	904,034	869,247	96.2	705,984
F	Fayetteville	S11	495,227	476,026	96.1	376,829
		S12	860,221	829,259	96.4	677,953
G	North	S13	886,695	848,726	95.7	692,452
		S14	803,096	775,715	96.6	642,329
		S17	913,691	881,743	96.5	746,262
H	Stratton	S15	948,914	908,703	95.8	741,907
		S16	476,703	459,319	96.4	378,918

**Table 4.** Type II ANOVA. Shannon diversity index (within-sample variation) was used as the response variable. Predictors are shown in the first column. Predictors were ordered by their absolute pairwise Pearson’s correlation coefficient with Shannon diversity index.

	Pearson’s Correlation with Shannon Diversity	Sum Sq	Df	F-Value	Pr (>F)	
SO4-S	−0.657	0.248172	1	446.0492	0.002234	**
Fe	−0.347	0.012271	1	22.05459	0.042474	*
Mg	−0.294	0.000161	1	0.290031	0.644121	
ECEC	−0.274	0.035436	1	63.69047	0.015341	*
Ca	−0.241	0.036469	1	65.54779	0.014916	*
B	−0.195	0.058488	1	105.1231	0.009379	**
Mn	0.187	0.016118	1	28.97011	0.032828	*
pH	−0.135	0.007749	1	13.92685	0.064893	.
Cu	−0.121	0.120172	1	215.99)79	0.004598	**
Zn	−0.086	0.012036	1	21.63242	0.04325	*
K	−0.063	0.001380	1	2.485264	0.255624	
P	−0.043	0.249761	1	418.9144	0.00222	**
Rotation		0.001880	1	3.390498	0.206919	
Till		0.000490	1	13.46163	0.066902	.
Residuals		0.001110	2			

P = phosphorus, K = potassium, Ca = calcium, Mg = magnesium, SO4-S = sulfate-sulfur, Zn = zinc, Fe = iron, Mn = manganese, Cu = copper, and B = boron. Rotation: no rotation (rice monoculture) or crop rotation. Till = cultivation method: tillage or no-till. Asterisks represent significance level.

* *p* > 0.05;

** *p* > 0.005.

Period (.) = marginally significant.

## Data Availability

The 16S rRNA sequencing data presented in this study is available at the NCBI Sequence Read Archive (SRA) (http://www.ncbi.nlm.nih.gov/sra) (accessed on 15 December 2021) under accession number PRJNA782652.
