# Supplementary material for: 16S rRNA Gene-Based Metagenomic Analysis of Rhizosphere Soil Bacteria in Arkansas Rice Crop Fields
Source: Agronomy (Basel). Author manuscript; Available in PMC 2026 Jun 13. (PMC13262776; doi:10.3390/agronomy12010222)
Supplement: Supplementary Material [file NIHMS2182077-supplement-Supplementary_Material.pdf]

Table S1: Class level diversity for the eight locations analyzed (17 samples). See table 1 for location details.

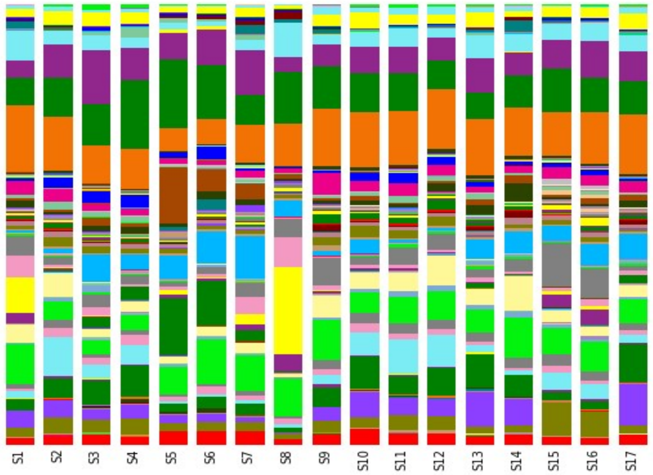

		Total	S1	S2	S3	S4	S5	S6	S7	S8	S9	S10	S11	S12	S13	S14	S15	S16	S17
Legend	Taxonomy	%	%	%	%	%	%	%	%	%	%	%	%	%	%	%	%	%	%
	k__Bacteria;p__Proteobacteria;c__Alphaproteobacteria	10.90%	15.00%	12.30%	8.60%	9.00%	5.30%	5.70%	8.70%	9.60%	12.90%	12.50%	12.30%	13.70%	12.70%	11.00%	10.00%	11.80%	13.50%
	k__Bacteria;p__Proteobacteria;c__Betaproteobacteria	9.30%	6.40%	8.90%	9.50%	15.70%	15.50%	12.30%	6.80%	11.60%	9.60%	9.00%	8.40%	6.40%	6.10%	7.10%	9.80%	7.80%	7.70%
	k__Bacteria;p__Proteobacteria;c__Deltaproteobacteria	6.80%	4.00%	7.40%	12.30%	7.40%	6.10%	8.10%	9.90%	3.50%	5.00%	5.80%	6.20%	5.40%	7.60%	5.40%	6.60%	8.20%	6.80%
	k__Bacteria;p__Actinobacteria;c__Actinobacteria	6.50%	9.20%	4.10%	3.00%	3.30%	6.20%	10.20%	8.00%	8.50%	9.10%	4.70%	6.00%	6.50%	5.20%	9.00%	6.10%	6.70%	5.30%
	k__Bacteria;p__Acidobacteria;c__Solibacteres	4.40%	2.60%	4.50%	5.40%	7.00%	1.90%	2.70%	2.70%	0.90%	4.40%	7.40%	4.10%	6.30%	7.50%	4.90%	2.10%	1.90%	8.80%
	k__Bacteria;p__Chloroflexi;c__Anaerolineae	3.90%	0.50%	1.10%	6.20%	3.40%	5.20%	7.20%	9.60%	3.80%	1.10%	3.10%	0.60%	0.60%	4.50%	4.90%	3.40%	4.70%	5.70%
	k__Bacteria;p__Bacteroidetes;c__[Saprospirae]	3.50%	4.30%	1.90%	2.70%	2.30%	1.20%	1.70%	3.20%	4.20%	6.00%	1.90%	3.80%	3.60%	1.80%	2.70%	9.90%	6.90%	1.20%
	k__Bacteria;p__Proteobacteria;c__Gammaproteobacteria	3.50%	6.60%	3.10%	3.20%	2.30%	1.70%	1.00%	2.50%	7.70%	2.20%	3.50%	4.10%	1.90%	5.20%	3.80%	3.40%	4.10%	3.60%
	k__Bacteria;p__Acidobacteria;c__Acidobacteriia	3.40%	3.90%	3.70%	2.20%	3.20%	1.90%	1.90%	1.00%	0.20%	3.10%	5.60%	3.70%	4.00%	7.80%	6.00%	0.10%	0.10%	9.40%
	k__Bacteria;p__Actinobacteria;c__Thermoleophilia	3.40%	4.20%	5.40%	1.60%	2.40%	1.60%	2.00%	2.20%	0.90%	4.80%	3.60%	4.20%	6.70%	3.50%	7.70%	2.60%	2.10%	2.60%
	k__Bacteria;p__Acidobacteria;c__[Chloracidobacteria]	3.20%	1.60%	8.70%	3.30%	4.40%	0.40%	0.40%	0.80%	0.90%	2.10%	5.20%	7.70%	7.30%	1.40%	1.00%	3.90%	3.40%	1.70%
	k__Bacteria;p__Acidobacteria;c__Acidobacteria-6	2.90%	2.00%	3.70%	3.10%	3.60%	1.80%	2.10%	1.90%	1.20%	2.70%	2.40%	3.50%	3.10%	1.50%	1.70%	7.50%	5.70%	1.70%
	Unassigned;Other;Other	2.40%	1.60%	2.10%	2.40%	2.00%	3.00%	3.00%	3.20%	1.30%	2.70%	3.50%	2.70%	2.60%	2.10%	2.40%	1.90%	1.60%	2.30%
	k__Bacteria;p__Verrucomicrobia;c__[Pedosphaerae]	2.40%	1.90%	3.20%	3.20%	2.80%	1.50%	1.70%	2.10%	0.60%	2.80%	3.50%	2.30%	1.90%	3.50%	1.20%	2.30%	2.30%	3.80%
	k__Bacteria;p__Bacteroidetes;c__Bacteroidia	2.10%	0.00%	0.00%	2.30%	2.90%	13.10%	10.30%	2.70%	0.40%	0.00%	0.10%	0.00%	0.00%	0.90%	0.30%	0.00%	0.10%	1.70%
	k__Bacteria;p__Gemmatimonadetes;c__Gemmatimonadetes	2.10%	3.00%	2.90%	1.50%	1.80%	1.10%	1.10%	1.50%	0.90%	4.90%	2.30%	2.90%	2.50%	1.30%	1.60%	2.40%	2.30%	2.40%
	k__Bacteria;p__Bacteroidetes;c__Flavobacteriia	2.00%	8.10%	0.10%	0.20%	0.00%	1.10%	0.40%	2.40%	19.80%	0.10%	0.00%	0.00%	0.00%	0.10%	0.00%	0.80%	0.70%	0.00%
	k__Bacteria;p__Firmicutes;c__Clostridia	2.00%	0.40%	0.40%	1.80%	0.80%	12.60%	4.90%	3.70%	0.90%	0.50%	0.90%	0.20%	0.50%	1.20%	1.60%	0.60%	1.20%	1.30%
	k__Bacteria;p__Bacteroidetes;c__Sphingobacteriia	1.70%	5.00%	0.50%	1.70%	0.40%	0.70%	0.70%	3.90%	6.80%	1.20%	0.80%	0.80%	0.70%	1.20%	0.60%	0.90%	1.70%	0.90%
	k__Bacteria;p__Actinobacteria;c__Acidimicrobiia	1.60%	1.10%	1.80%	0.90%	1.10%	0.60%	0.60%	1.10%	0.60%	2.10%	2.30%	2.30%	2.10%	1.90%	2.50%	2.60%	2.10%	1.60%
	k__Bacteria;p__Firmicutes;c__Bacilli	1.40%	1.70%	0.70%	1.40%	1.60%	0.90%	2.00%	1.20%	0.50%	0.70%	1.10%	0.90%	1.80%	2.20%	4.20%	0.50%	0.60%	1.40%
	k__Bacteria;p__Nitrospirae;c__Nitrospira	1.40%	0.50%	2.40%	2.60%	2.80%	1.30%	2.80%	0.60%	0.20%	0.60%	1.60%	2.20%	1.60%	1.00%	0.80%	0.90%	1.20%	0.50%
	k__Bacteria;p__Acidobacteria;c__iii1-8	1.30%	0.40%	2.20%	1.50%	2.30%	1.00%	1.70%	0.90%	1.20%	1.30%	2.00%	1.40%	1.20%	0.90%	0.50%	1.50%	0.80%	1.20%
	k__Bacteria;p__Chloroflexi;c__Chloroflexi	1.20%	0.70%	2.20%	1.30%	1.20%	1.00%	0.80%	0.80%	0.70%	1.80%	2.70%	1.90%	2.10%	0.30%	0.70%	0.90%	1.10%	0.60%
	k__Bacteria;p__Bacteroidetes;c__Cytophagia	1.00%	2.40%	0.70%	0.30%	0.10%	0.70%	0.40%	1.30%	3.80%	0.60%	0.20%	0.50%	0.10%	0.20%	0.10%	2.90%	3.40%	0.10%
	k__Bacteria;p__Chloroflexi;c__Ellin6529	0.80%	0.90%	1.20%	0.80%	0.80%	0.60%	0.90%	0.70%	0.40%	1.20%	0.70%	0.70%	0.70%	0.70%	1.40%	1.10%	1.10%	0.60%
	k__Bacteria;p__Chloroflexi;c__TK10	0.80%	0.60%	1.00%	0.50%	0.60%	0.30%	0.30%	0.20%	0.10%	2.10%	1.20%	1.30%	2.30%	0.70%	0.60%	0.50%	0.70%	0.80%
	k__Bacteria;p__Gemmatimonadetes;c__Gemm-1	0.80%	0.60%	1.70%	1.20%	1.40%	0.40%	0.30%	0.60%	0.30%	0.50%	0.90%	1.50%	1.20%	1.20%	0.50%	0.80%	1.00%	0.30%
	k__Bacteria;p__Verrucomicrobia;c__[Spartobacteria]	0.80%	0.90%	1.00%	0.50%	0.70%	0.10%	0.10%	0.50%	0.30%	1.60%	1.20%	1.50%	1.90%	1.00%	1.00%	0.20%	0.20%	1.20%
	k__Bacteria;p__Actinobacteria;c__MB-A2-108	0.70%	0.20%	1.00%	0.30%	0.50%	0.60%	0.50%	0.40%	0.10%	0.70%	0.80%	1.40%	1.40%	0.90%	1.40%	0.90%	0.60%	0.50%
	k__Bacteria;p__TM7;c__TM7-1	0.70%	1.70%	0.70%	0.10%	0.10%	0.20%	0.10%	2.10%	0.60%	0.70%	0.30%	0.20%	0.60%	0.50%	2.70%	0.40%	0.30%	0.30%
	k__Bacteria;p__Chloroflexi;c__C0119	0.50%	0.50%	0.50%	0.40%	0.30%	0.50%	0.60%	0.40%	0.50%	1.30%	0.50%	0.30%	0.50%	0.50%	0.80%	0.40%	0.50%	0.50%
	k__Bacteria;p__Chloroflexi;c__Thermomicrobia	0.50%	0.70%	0.60%	0.20%	0.30%	0.20%	0.30%	0.60%	0.90%	0.70%	0.20%	0.40%	0.70%	0.10%	0.20%	1.20%	1.90%	0.10%
	k__Bacteria;p__Planctomycetes;c__Planctomycetia	0.50%	0.40%	0.60%	0.30%	0.30%	0.30%	0.40%	0.40%	0.20%	0.70%	0.70%	0.60%	0.90%	0.70%	0.70%	0.40%	0.60%	0.60%
	k__Bacteria;p__Chloroflexi;c__Ktedonobacteria	0.40%	0.30%	0.30%	0.00%	0.10%	0.30%	0.30%	0.00%	0.00%	1.70%	0.30%	0.10%	0.30%	0.80%	1.60%	0.00%	0.00%	1.10%
	k__Bacteria;p__Elusimicrobia;c__Elusimicrobia	0.40%	0.30%	0.60%	0.30%	0.20%	0.10%	0.10%	0.50%	0.10%	0.20%	0.60%	0.70%	0.40%	0.50%	0.10%	0.50%	0.40%	0.70%
	k__Bacteria;p__WS3;c__PRR-12	0.40%	0.00%	0.40%	1.10%	0.80%	0.30%	0.30%	0.10%	0.00%	0.00%	0.60%	0.60%	0.40%	0.40%	0.20%	0.30%	0.50%	0.30%
	k__Bacteria;p__Chlamydiae;c__Chlamydiia	0.30%	0.40%	0.40%	0.50%	0.30%	0.00%	0.00%	0.10%	0.00%	0.10%	0.30%	0.80%	0.40%	0.70%	0.60%	0.10%	0.30%	0.30%
	k__Bacteria;p__Chlorobi;c__BSV26	0.30%	0.00%	0.00%	1.60%	0.30%	0.20%	0.30%	0.20%	0.00%	0.00%	0.20%	0.10%	0.00%	0.80%	0.40%	0.00%	0.00%	0.30%
	k__Bacteria;p__Cyanobacteria;c__Chloroplast	0.30%	0.20%	0.10%	0.10%	0.00%	0.40%	0.50%	0.90%	0.80%	0.60%	0.20%	0.10%	0.10%	0.10%	0.20%	0.20%	0.60%	0.20%
	k__Bacteria;p__Gemmatimonadetes;c__Gemm-5	0.30%	0.10%	0.10%	0.10%	0.10%	0.40%	0.60%	0.40%	0.10%	0.20%	0.10%	0.00%	0.10%	0.20%	0.10%	1.50%	0.90%	0.20%
	k__Bacteria;p__Spirochaetes;c__Spirochaetes	0.30%	0.00%	0.00%	0.60%	2.30%	0.90%	0.50%	0.80%	0.10%	0.00%	0.10%	0.00%	0.00%	0.40%	0.00%	0.00%	0.00%	0.20%
	k__Bacteria;p__TM7;c__TM7-3	0.30%	0.20%	0.00%	0.00%	0.10%	0.50%	0.70%	1.10%	1.90%	0.10%	0.00%	0.00%	0.00%	0.10%	0.70%	0.00%	0.00%	0.10%
	k__Bacteria;p__Verrucomicrobia;c__Opitutae	0.30%	0.40%	0.10%	0.80%	0.20%	0.40%	0.30%	0.60%	0.20%	0.30%	0.10%	0.20%	0.10%	0.10%	0.00%	0.50%	0.40%	0.20%
	k__Bacteria;p__Acidobacteria;c__Acidobacteria-5	0.20%	0.10%	0.40%	0.10%	0.10%	0.00%	0.00%	0.00%	0.00%	0.10%	0.20%	0.40%	0.60%	0.10%	0.10%	0.20%	0.20%	0.10%
	k__Bacteria;p__Acidobacteria;c__BPC102	0.20%	0.00%	0.00%	0.40%	0.30%	0.20%	0.30%	0.30%	0.10%	0.00%	0.20%	0.00%	0.00%	0.20%	0.20%	0.00%	0.10%	0.30%
	k__Bacteria;p__Acidobacteria;c__Holophagae	0.20%	0.00%	0.00%	0.10%	0.80%	0.40%	0.80%	0.20%	0.00%	0.00%	0.10%	0.20%	0.00%	0.10%	0.10%	0.00%	0.00%	0.10%
	k__Bacteria;p__Acidobacteria;c__S035	0.20%	0.00%	0.30%	0.30%	0.40%	0.10%	0.10%	0.00%	0.00%	0.00%	0.30%	0.30%	0.40%	0.10%	0.00%	0.10%	0.30%	0.00%
	k__Bacteria;p__Acidobacteria;c__Sva0725	0.20%	0.10%	0.30%	0.40%	0.30%	0.00%	0.00%	0.00%	0.00%	0.60%	0.20%	0.20%	0.20%	0.10%	0.10%	0.40%	0.40%	0.20%
	k__Bacteria;p__Chlorobi;c__	0.20%	0.30%	0.20%	0.10%	0.20%	0.00%	0.00%	0.30%	0.10%	0.20%	0.10%	0.20%	0.20%	0.20%	0.00%	0.10%	0.10%	0.20%
	k__Bacteria;p__Chloroflexi;c__S085	0.20%	0.20%	0.30%	0.20%	0.20%	0.10%	0.10%	0.00%	0.00%	0.10%	0.20%	0.40%	0.40%	0.30%	0.20%	0.30%	0.40%	0.10%
	k__Bacteria;p__Chloroflexi;c__TK17	0.20%	0.10%	0.20%	0.30%	0.20%	0.10%	0.10%	0.10%	0.10%	0.20%	0.20%	0.20%	0.30%	0.30%	0.20%	0.30%	0.20%	0.20%
	k__Bacteria;p__Fibrobacteres;c__Fibrobacteria	0.20%	0.10%	0.10%	0.10%	0.00%	0.20%	0.00%	1.70%	0.70%	0.10%	0.00%	0.10%	0.00%	0.10%	0.00%	0.00%	0.00%	0.00%
	k__Bacteria;p__Fibrobacteres;c__TG3	0.20%	0.00%	0.00%	0.10%	0.00%	0.60%	2.40%	0.10%	0.00%	0.00%	0.00%	0.00%	0.00%	0.00%	0.10%	0.00%	0.00%	0.10%
	k__Bacteria;p__OD1;c__SM2F11	0.20%	0.40%	0.20%	0.10%	0.10%	0.00%	0.10%	0.20%	0.00%	0.00%	0.10%	0.20%	0.30%	0.10%	0.20%	0.50%	0.20%	0.10%
	k__Bacteria;p__Planctomycetes;c__Phycisphaerae	0.20%	0.10%	0.10%	0.20%	0.20%	0.00%	0.00%	0.10%	0.00%	0.10%	0.20%	0.20%	0.10%	0.30%	0.20%	0.30%	0.30%	0.30%
	k__Bacteria;p__Acidobacteria;c__DA052	0.10%	0.00%	0.10%	0.00%	0.10%	0.00%	0.00%	0.00%	0.00%	0.00%	0.10%	0.20%	0.20%	0.60%	0.10%	0.00%	0.00%	0.10%
	k__Bacteria;p__Acidobacteria;c__TM1	0.10%	0.10%	0.10%	0.10%	0.10%	0.00%	0.00%	0.00%	0.00%	0.00%	0.10%	0.30%	0.10%	0.50%	0.00%	0.00%	0.00%	0.10%
	k__Bacteria;p__Actinobacteria;c__OPB41	0.10%	0.00%	0.00%	0.50%	0.20%	0.20%	0.30%	0.20%	0.10%	0.00%	0.20%	0.00%	0.00%	0.10%	0.30%	0.00%	0.10%	0.10%

	k__Bacteria;p__Actinobacteria;c__Rubrobacteria	0.10%	0.00%	0.10%	0.10%	0.20%	0.00%	0.00%	0.00%	0.10%	0.00%	0.00%	0.00%	0.00%	0.00%	0.00%	0.40%	0.60%	0.00%
	k__Bacteria;p__Armatimonadetes;c__0319-6E2	0.10%	0.10%	0.10%	0.00%	0.00%	0.00%	0.00%	0.00%	0.00%	0.20%	0.00%	0.10%	0.10%	0.00%	0.00%	0.10%	0.10%	0.00%
	k__Bacteria;p__Armatimonadetes;c__Chthonomonadetes	0.10%	0.00%	0.10%	0.10%	0.10%	0.00%	0.00%	0.00%	0.00%	0.10%	0.20%	0.20%	0.20%	0.20%	0.10%	0.10%	0.10%	0.20%
	k__Bacteria;p__Armatimonadetes;c__[Fimbriimonadia]	0.10%	0.10%	0.20%	0.10%	0.10%	0.00%	0.00%	0.00%	0.00%	0.10%	0.10%	0.20%	0.10%	0.10%	0.10%	0.20%	0.10%	0.10%
	k__Bacteria;p__Chlorobi;c__Ignavibacteria	0.10%	0.00%	0.00%	0.50%	0.20%	0.10%	0.10%	0.30%	0.00%	0.00%	0.00%	0.00%	0.00%	0.10%	0.10%	0.00%	0.00%	0.30%
	k__Bacteria;p__Chlorobi;c__OPB56	0.10%	0.00%	0.00%	0.10%	0.00%	0.20%	0.10%	0.10%	0.10%	0.10%	0.00%	0.00%	0.00%	0.20%	0.00%	0.10%	0.10%	0.20%
	k__Bacteria;p__Chloroflexi;c__Dehalococcoidetes	0.10%	0.00%	0.00%	0.10%	0.10%	0.30%	0.50%	0.30%	0.10%	0.00%	0.10%	0.00%	0.00%	0.30%	0.20%	0.00%	0.10%	0.40%
	k__Bacteria;p__Chloroflexi;c__Gitt-GS-136	0.10%	0.00%	0.10%	0.00%	0.10%	0.00%	0.00%	0.10%	0.10%	0.10%	0.10%	0.10%	0.10%	0.10%	0.20%	0.10%	0.10%	0.10%
	k__Bacteria;p__Cyanobacteria;c__4C0d-2	0.10%	0.20%	0.10%	0.10%	0.10%	0.00%	0.00%	0.10%	0.00%	0.10%	0.10%	0.20%	0.10%	0.20%	0.10%	0.10%	0.10%	0.20%
	k__Bacteria;p__Cyanobacteria;c__ML635J-21	0.10%	0.20%	0.20%	0.10%	0.10%	0.00%	0.00%	0.20%	0.00%	0.10%	0.10%	0.20%	0.10%	0.10%	0.20%	0.10%	0.10%	0.20%
	k__Bacteria;p__Cyanobacteria;c__Synechococcophycideae	0.10%	0.00%	0.00%	0.00%	0.00%	0.00%	0.10%	0.20%	0.00%	0.10%	0.20%	0.00%	0.00%	0.00%	0.00%	0.20%	0.80%	0.00%
	k__Bacteria;p__Elusimicrobia;c__Endomicrobia	0.10%	0.00%	0.00%	0.20%	0.20%	0.00%	0.00%	0.10%	0.00%	0.00%	0.10%	0.00%	0.00%	0.10%	0.10%	0.00%	0.00%	0.10%
	k__Bacteria;p__FCPU426;c__	0.10%	0.10%	0.10%	0.00%	0.00%	0.00%	0.00%	0.00%	0.00%	0.00%	0.10%	0.40%	0.00%	0.10%	0.00%	0.00%	0.00%	0.10%
	k__Bacteria;p__Fibrobacteres;c__	0.10%	0.00%	0.00%	0.00%	0.00%	1.40%	0.20%	0.10%	0.00%	0.00%	0.00%	0.00%	0.00%	0.00%	0.00%	0.00%	0.00%	0.00%
	k__Bacteria;p__Gemmatimonadetes;c__	0.10%	0.00%	0.00%	0.00%	0.00%	0.00%	0.00%	0.00%	0.00%	0.00%	0.00%	0.00%	0.00%	0.00%	0.00%	0.90%	0.60%	0.00%
	k__Bacteria;p__OD1;c__	0.10%	0.10%	0.10%	0.20%	0.10%	0.00%	0.10%	0.10%	0.00%	0.00%	0.00%	0.10%	0.10%	0.00%	0.00%	0.10%	0.00%	0.00%
	k__Bacteria;p__OD1;c__ABY1	0.10%	0.10%	0.00%	0.20%	0.10%	0.00%	0.00%	0.10%	0.00%	0.00%	0.00%	0.10%	0.00%	0.20%	0.00%	0.00%	0.00%	0.00%
	k__Bacteria;p__OD1;c__ZB2	0.10%	0.10%	0.10%	0.00%	0.00%	0.00%	0.10%	0.40%	0.00%	0.00%	0.00%	0.10%	0.10%	0.10%	0.00%	0.00%	0.00%	0.00%
	k__Bacteria;p__OP3;c__koll11	0.10%	0.10%	0.10%	0.10%	0.10%	0.00%	0.00%	0.00%	0.00%	0.00%	0.10%	0.20%	0.10%	0.10%	0.00%	0.10%	0.00%	0.00%
	k__Bacteria;p__Proteobacteria;c__Epsilonproteobacteria	0.10%	0.00%	0.00%	0.00%	0.00%	0.70%	0.60%	0.10%	0.00%	0.00%	0.00%	0.00%	0.00%	0.00%	0.20%	0.00%	0.00%	0.00%
	k__Bacteria;p__TM6;c__SJA-4	0.10%	0.10%	0.10%	0.10%	0.10%	0.00%	0.00%	0.00%	0.00%	0.00%	0.10%	0.10%	0.00%	0.20%	0.20%	0.00%	0.00%	0.10%
	k__Bacteria;p__TM7;c__SC3	0.10%	0.10%	0.10%	0.00%	0.00%	0.00%	0.00%	0.20%	0.00%	0.20%	0.10%	0.00%	0.20%	0.10%	0.20%	0.30%	0.10%	0.20%
	k__Bacteria;p__Verrucomicrobia;c__Verrucomicrobiae	0.10%	0.20%	0.10%	0.00%	0.00%	0.10%	0.10%	0.20%	0.40%	0.10%	0.00%	0.00%	0.00%	0.00%	0.00%	0.20%	0.20%	0.00%
	k__Bacteria;p__WPS-2;c__	0.10%	0.10%	0.10%	0.00%	0.00%	0.00%	0.00%	0.00%	0.00%	0.40%	0.10%	0.10%	0.10%	0.20%	0.30%	0.00%	0.00%	0.40%
	k__Archaea;p__Crenarchaeota;c__MBGA	0.00%	0.00%	0.00%	0.00%	0.00%	0.00%	0.00%	0.00%	0.00%	0.00%	0.00%	0.00%	0.00%	0.00%	0.00%	0.00%	0.00%	0.00%
	k__Archaea;p__Crenarchaeota;c__MCG	0.00%	0.00%	0.00%	0.00%	0.00%	0.00%	0.00%	0.00%	0.00%	0.00%	0.00%	0.00%	0.00%	0.00%	0.00%	0.00%	0.00%	0.00%
	k__Archaea;p__Crenarchaeota;c__Thaumarchaeota	0.00%	0.00%	0.00%	0.00%	0.00%	0.00%	0.00%	0.00%	0.00%	0.00%	0.00%	0.00%	0.00%	0.00%	0.00%	0.00%	0.00%	0.00%
	k__Archaea;p__Euryarchaeota;c__DSEG	0.00%	0.00%	0.00%	0.00%	0.00%	0.00%	0.00%	0.00%	0.00%	0.00%	0.00%	0.00%	0.00%	0.00%	0.00%	0.00%	0.00%	0.00%
	k__Archaea;p__Euryarchaeota;c__Methanobacteria	0.00%	0.00%	0.00%	0.00%	0.00%	0.00%	0.00%	0.00%	0.00%	0.00%	0.00%	0.00%	0.00%	0.00%	0.00%	0.00%	0.00%	0.00%
	k__Archaea;p__Euryarchaeota;c__Methanomicrobia	0.00%	0.00%	0.00%	0.00%	0.00%	0.00%	0.00%	0.00%	0.00%	0.00%	0.00%	0.00%	0.00%	0.00%	0.00%	0.00%	0.00%	0.00%
	k__Archaea;p__Euryarchaeota;c__Thermoplasmata	0.00%	0.00%	0.00%	0.00%	0.00%	0.00%	0.00%	0.00%	0.00%	0.00%	0.00%	0.00%	0.00%	0.00%	0.00%	0.00%	0.00%	0.00%
	k__Archaea;p__[Parvarchaeota];c__[Micrarchaea]	0.00%	0.00%	0.00%	0.00%	0.00%	0.00%	0.00%	0.00%	0.00%	0.00%	0.00%	0.00%	0.00%	0.00%	0.00%	0.00%	0.00%	0.00%
	k__Archaea;p__[Parvarchaeota];c__[Parvarchaea]	0.00%	0.00%	0.00%	0.00%	0.00%	0.00%	0.00%	0.00%	0.00%	0.00%	0.00%	0.00%	0.00%	0.00%	0.00%	0.00%	0.00%	0.00%
	k__Bacteria;Other;Other	0.00%	0.00%	0.00%	0.00%	0.00%	0.00%	0.00%	0.00%	0.00%	0.00%	0.00%	0.00%	0.00%	0.00%	0.00%	0.00%	0.00%	0.00%
	k__Bacteria;p__;c__	0.00%	0.00%	0.00%	0.00%	0.00%	0.00%	0.00%	0.00%	0.00%	0.00%	0.00%	0.00%	0.00%	0.00%	0.00%	0.00%	0.00%	0.00%
	k__Bacteria;p__AC1;Other	0.00%	0.00%	0.00%	0.00%	0.00%	0.00%	0.00%	0.00%	0.00%	0.00%	0.00%	0.00%	0.00%	0.00%	0.00%	0.00%	0.00%	0.00%
	k__Bacteria;p__AC1;c__HDBW-WB69	0.00%	0.00%	0.00%	0.00%	0.00%	0.00%	0.00%	0.00%	0.00%	0.00%	0.00%	0.00%	0.00%	0.00%	0.00%	0.00%	0.00%	0.00%
	k__Bacteria;p__AC1;c__SHA-114	0.00%	0.00%	0.00%	0.00%	0.00%	0.00%	0.00%	0.00%	0.00%	0.00%	0.00%	0.00%	0.00%	0.00%	0.00%	0.00%	0.00%	0.00%
	k__Bacteria;p__AD3;c__	0.00%	0.00%	0.00%	0.00%	0.00%	0.00%	0.00%	0.00%	0.00%	0.00%	0.00%	0.00%	0.00%	0.00%	0.00%	0.00%	0.00%	0.00%
	k__Bacteria;p__AD3;c__ABS-6	0.00%	0.00%	0.00%	0.00%	0.00%	0.00%	0.00%	0.00%	0.00%	0.00%	0.00%	0.00%	0.00%	0.20%	0.20%	0.00%	0.00%	0.10%
	k__Bacteria;p__AD3;c__JG37-AG-4	0.00%	0.00%	0.00%	0.00%	0.00%	0.00%	0.00%	0.00%	0.00%	0.00%	0.10%	0.00%	0.00%	0.10%	0.10%	0.00%	0.00%	0.10%
	k__Bacteria;p__Acidobacteria;Other	0.00%	0.00%	0.00%	0.00%	0.00%	0.00%	0.00%	0.00%	0.00%	0.00%	0.00%	0.00%	0.00%	0.00%	0.00%	0.00%	0.00%	0.00%
	k__Bacteria;p__Acidobacteria;c__	0.00%	0.00%	0.10%	0.10%	0.20%	0.00%	0.00%	0.00%	0.00%	0.00%	0.00%	0.10%	0.00%	0.00%	0.00%	0.00%	0.00%	0.00%
	k__Bacteria;p__Acidobacteria;c__AT-s54	0.00%	0.00%	0.00%	0.00%	0.00%	0.00%	0.00%	0.00%	0.00%	0.00%	0.00%	0.00%	0.00%	0.00%	0.00%	0.00%	0.00%	0.00%
	k__Bacteria;p__Acidobacteria;c__EC1113	0.00%	0.00%	0.10%	0.00%	0.00%	0.00%	0.00%	0.00%	0.00%	0.00%	0.00%	0.00%	0.00%	0.00%	0.00%	0.00%	0.00%	0.00%
	k__Bacteria;p__Acidobacteria;c__OS-K	0.00%	0.00%	0.00%	0.00%	0.00%	0.00%	0.00%	0.00%	0.00%	0.00%	0.00%	0.00%	0.00%	0.00%	0.00%	0.00%	0.00%	0.00%
	k__Bacteria;p__Acidobacteria;c__PAUC37f	0.00%	0.00%	0.00%	0.10%	0.10%	0.00%	0.00%	0.00%	0.00%	0.00%	0.00%	0.10%	0.10%	0.00%	0.00%	0.00%	0.00%	0.00%
	k__Bacteria;p__Acidobacteria;c__RB25	0.00%	0.00%	0.00%	0.10%	0.00%	0.00%	0.00%	0.00%	0.00%	0.00%	0.00%	0.00%	0.00%	0.00%	0.00%	0.10%	0.10%	0.00%
	k__Bacteria;p__Actinobacteria;Other	0.00%	0.00%	0.00%	0.00%	0.00%	0.00%	0.00%	0.00%	0.00%	0.00%	0.00%	0.00%	0.00%	0.00%	0.00%	0.00%	0.00%	0.00%
	k__Bacteria;p__Actinobacteria;c__	0.00%	0.00%	0.00%	0.00%	0.00%	0.00%	0.00%	0.00%	0.00%	0.00%	0.00%	0.00%	0.00%	0.00%	0.00%	0.00%	0.00%	0.00%
	k__Bacteria;p__Actinobacteria;c__KIST-JJY010	0.00%	0.00%	0.00%	0.00%	0.00%	0.00%	0.00%	0.00%	0.00%	0.00%	0.00%	0.00%	0.00%	0.00%	0.00%	0.00%	0.00%	0.00%
	k__Bacteria;p__Actinobacteria;c__Nitriliruptoria	0.00%	0.00%	0.00%	0.00%	0.00%	0.00%	0.00%	0.00%	0.00%	0.00%	0.00%	0.00%	0.00%	0.00%	0.00%	0.10%	0.00%	0.00%
	k__Bacteria;p__Armatimonadetes;c__Armatimonadia	0.00%	0.00%	0.00%	0.00%	0.00%	0.00%	0.00%	0.00%	0.00%	0.00%	0.00%	0.00%	0.00%	0.00%	0.00%	0.00%	0.00%	0.00%
	k__Bacteria;p__Armatimonadetes;c__OPB50	0.00%	0.00%	0.00%	0.00%	0.00%	0.00%	0.00%	0.00%	0.00%	0.00%	0.00%	0.00%	0.00%	0.00%	0.00%	0.00%	0.00%	0.00%
	k__Bacteria;p__Armatimonadetes;c__SHA-37	0.00%	0.00%	0.00%	0.00%	0.00%	0.00%	0.00%	0.00%	0.00%	0.00%	0.00%	0.00%	0.00%	0.00%	0.00%	0.00%	0.00%	0.00%
	k__Bacteria;p__Armatimonadetes;c__SJA-176	0.00%	0.00%	0.00%	0.10%	0.10%	0.00%	0.00%	0.10%	0.00%	0.00%	0.00%	0.00%	0.00%	0.00%	0.10%	0.00%	0.00%	0.00%
	k__Bacteria;p__BHI80-139;c__	0.00%	0.00%	0.00%	0.00%	0.00%	0.00%	0.00%	0.00%	0.00%	0.00%	0.00%	0.00%	0.10%	0.00%	0.00%	0.10%	0.00%	0.00%
	k__Bacteria;p__BRC1;c__	0.00%	0.00%	0.00%	0.00%	0.00%	0.00%	0.00%	0.00%	0.00%	0.00%	0.00%	0.00%	0.00%	0.00%	0.00%	0.00%	0.00%	0.00%
	k__Bacteria;p__BRC1;c__NPL-UPA2	0.00%	0.00%	0.00%	0.00%	0.00%	0.00%	0.00%	0.00%	0.00%	0.00%	0.00%	0.00%	0.00%	0.00%	0.00%	0.00%	0.00%	0.00%
	k__Bacteria;p__BRC1;c__PRR-11	0.00%	0.00%	0.00%	0.10%	0.10%	0.00%	0.00%	0.00%	0.00%	0.00%	0.10%	0.00%	0.00%	0.10%	0.00%	0.00%	0.00%	0.10%
	k__Bacteria;p__Bacteroidetes;Other	0.00%	0.00%	0.00%	0.00%	0.00%	0.00%	0.00%	0.00%	0.00%	0.00%	0.00%	0.00%	0.00%	0.00%	0.00%	0.00%	0.00%	0.00%
	k__Bacteria;p__Bacteroidetes;c__At12OctB3	0.00%	0.00%	0.00%	0.00%	0.00%	0.00%	0.00%	0.00%	0.00%	0.10%	0.00%	0.00%	0.00%	0.00%	0.00%	0.00%	0.00%	0.00%
	k__Bacteria;p__Bacteroidetes;c__BME43	0.00%	0.00%	0.00%	0.00%	0.00%	0.00%	0.00%	0.00%	0.00%	0.00%	0.00%	0.00%	0.00%	0.00%	0.00%	0.00%	0.00%	0.00%
	k__Bacteria;p__Bacteroidetes;c__SM1A07	0.00%	0.00%	0.00%	0.00%	0.00%	0.00%	0.00%	0.00%	0.00%	0.00%	0.00%	0.00%	0.00%	0.00%	0.00%	0.00%	0.00%	0.00%
	k__Bacteria;p__Bacteroidetes;c__VC2_1_Bac22	0.00%	0.00%	0.00%	0.20%	0.00%	0.00%	0.00%	0.00%	0.00%	0.00%	0.00%	0.00%	0.00%	0.00%	0.00%	0.00%	0.00%	0.00%
	k__Bacteria;p__Bacteroidetes;c__[Rhodothermi]	0.00%	0.00%	0.00%	0.00%	0.00%	0.00%	0.00%	0.00%	0.00%	0.00%	0.00%	0.00%	0.00%	0.00%	0.00%	0.10%	0.10%	0.00%
	k__Bacteria;p__Chlorobi;c__Chlorobia	0.00%	0.00%	0.00%	0.00%	0.00%	0.00%	0.00%	0.00%	0.00%	0.00%	0.00%	0.00%	0.00%	0.00%	0.00%	0.00%	0.00%	0.00%
	k__Bacteria;p__Chlorobi;c__SJA-28	0.00%	0.00%	0.00%	0.10%	0.00%	0.00%	0.00%	0.00%	0.00%	0.00%	0.00%	0.00%	0.00%	0.20%	0.00%	0.00%	0.00%	0.00%
	k__Bacteria;p__Chloroflexi;Other	0.00%	0.00%	0.00%	0.00%	0.00%	0.00%	0.00%	0.00%	0.00%	0.00%	0.00%	0.00%	0.00%	0.00%	0.00%	0.00%	0.00%	0.00%
	k__Bacteria;p__Chloroflexi;c__	0.00%	0.00%	0.10%	0.00%	0.00%	0.10%	0.10%	0.00%	0.00%	0.10%	0.10%	0.00%	0.10%	0.00%	0.00%	0.10%	0.10%	0.00%
	k__Bacteria;p__Chloroflexi;c__MVP-21	0.00%	0.00%	0.00%	0.00%	0.00%	0.00%	0.00%	0.00%	0.00%	0.00%	0.00%	0.00%	0.00%	0.00%	0.00%	0.00%	0.00%	0.00%
	k__Bacteria;p__Chloroflexi;c__P2-11E	0.00%	0.00%	0.10%	0.00%	0.10%	0.00%	0.00%	0.00%	0.00%	0.00%	0.20%	0.10%	0.00%	0.00%	0.00%	0.00%	0.00%	0.10%
	k__Bacteria;p__Chloroflexi;c__SAR202	0.00%	0.00%	0.00%	0.00%	0.00%	0.00%	0.00%	0.00%	0.00%	0.00%	0.00%	0.00%	0.00%	0.00%	0.00%	0.00%	0.00%	0.00%
	k__Bacteria;p__Chloroflexi;c__SHA-26	0.00%	0.00%	0.00%	0.00%	0.00%	0.00%	0.10%	0.00%	0.00%	0.10%	0.10%	0.00%	0.10%	0.00%	0.10%	0.00%	0.00%	0.10%
	k__Bacteria;p__Chloroflexi;c__[Thermobacula]	0.00%	0.00%	0.00%	0.00%	0.00%	0.00%	0.00%	0.00%	0.00%	0.00%	0.00%	0.00%	0.00%	0.00%	0.00%	0.00%	0.00%	0.00%
	k__Bacteria;p__Cyanobacteria;Other	0.00%	0.00%	0.00%	0.00%	0.00%	0.00%	0.00%	0.00%	0.00%	0.00%	0.00%	0.00%	0.00%	0.00%	0.00%	0.00%	0.00%	0.00%
	k__Bacteria;p__Cyanobacteria;c__	0.00%	0.00%	0.00%	0.00%	0.00%	0.00%	0.00%	0.00%	0.00%	0.00%	0.00%	0.00%	0.00%	0.00%	0.00%	0.00%	0.00%	0.00%
	k__Bacteria;p__Cyanobacteria;c__Nostocophycideae	0.00%	0.00%	0.00%	0.00%	0.00%	0.00%	0.10%	0.10%	0.00%	0.00%	0.00%	0.00%	0.00%	0.00%	0.00%	0.00%	0.10%	0.00%
	k__Bacteria;p__Cyanobacteria;c__Oscillatoriophycideae	0.00%	0.00%	0.00%	0.00%	0.00%	0.00%	0.00%	0.00%	0.00%	0.00%	0.00%	0.00%	0.00%	0.00%	0.00%	0.00%	0.40%	0.00%
	k__Bacteria;p__Elusimicrobia;c__	0.00%	0.00%	0.00%	0.10%	0.00%	0.00%	0.00%	0.00%	0.00%	0.00%	0.00%	0.00%	0.00%	0.10%	0.00%	0.00%	0.00%	0.00%
	k__Bacteria;p__Elusimicrobia;c__OP2	0.00%	0.00%	0.00%	0.00%	0.00%	0.00%	0.00%	0.00%	0.00%	0.00%	0.00%	0.00%	0.00%	0.00%	0.00%	0.00%	0.00%	0.00%
	k__Bacteria;p__FBP;c__	0.00%	0.10%	0.00%	0.00%	0.00%	0.00%	0.00%	0.00%	0.00%	0.20%	0.00%	0.00%	0.00%	0.00%	0.00%	0.00%	0.00%	0.00%
	k__Bacteria;p__Fibrobacteres;c__B5-096	0.00%	0.00%	0.00%	0.00%	0.00%	0.00%	0.00%	0.00%	0.00%	0.00%	0.00%	0.00%	0.00%	0.00%	0.00%	0.00%	0.00%	0.00%
	k__Bacteria;p__Fibrobacteres;c__SBZC_2415	0.00%	0.00%	0.00%	0.00%	0.00%	0.00%	0.00%	0.00%	0.00%	0.00%	0.00%	0.00%	0.00%	0.00%	0.00%	0.00%	0.00%	0.00%
	k__Bacteria;p__Firmicutes;Other	0.00%	0.00%	0.00%	0.00%	0.00%	0.00%	0.00%	0.00%	0.00%	0.00%	0.00%	0.00%	0.00%	0.00%	0.00%	0.00%	0.00%	0.00%
	k__Bacteria;p__Firmicutes;c__Erysipelotrichi	0.00%	0.00%	0.00%	0.00%	0.00%	0.00%	0.00%	0.00%	0.00%	0.00%	0.00%	0.00%	0.00%	0.00%	0.00%	0.00%	0.00%	0.00%

k__Bacteria;p__Fusobacteria;c__Fusobacteriia	0.00%	0.00%	0.00%	0.00%	0.00%	0.00%	0.00%	0.00%	0.00%	0.00%	0.00%	0.00%	0.00%	0.00%	0.00%	0.00%	0.00%	0.00%
k__Bacteria;p__GAL15;c__	0.00%	0.00%	0.00%	0.00%	0.00%	0.00%	0.00%	0.00%	0.00%	0.00%	0.00%	0.00%	0.00%	0.10%	0.00%	0.00%	0.00%	0.00%
k__Bacteria;p__GN02;c__	0.00%	0.00%	0.00%	0.00%	0.00%	0.00%	0.00%	0.00%	0.00%	0.00%	0.00%	0.00%	0.00%	0.00%	0.00%	0.00%	0.00%	0.00%
k__Bacteria;p__GN02;c__3BR-5F	0.00%	0.00%	0.00%	0.00%	0.00%	0.00%	0.00%	0.00%	0.00%	0.00%	0.00%	0.00%	0.00%	0.00%	0.00%	0.00%	0.00%	0.00%
k__Bacteria;p__GN02;c__BB34	0.00%	0.00%	0.00%	0.00%	0.00%	0.00%	0.00%	0.00%	0.00%	0.00%	0.00%	0.00%	0.00%	0.00%	0.00%	0.00%	0.00%	0.00%
k__Bacteria;p__GN02;c__BD1-5	0.00%	0.00%	0.00%	0.00%	0.00%	0.00%	0.10%	0.00%	0.00%	0.00%	0.00%	0.00%	0.00%	0.00%	0.00%	0.00%	0.00%	0.00%
k__Bacteria;p__GN02;c__GKS2-174	0.00%	0.10%	0.10%	0.00%	0.00%	0.00%	0.00%	0.00%	0.00%	0.00%	0.00%	0.00%	0.10%	0.00%	0.00%	0.10%	0.00%	0.00%
k__Bacteria;p__GN02;c__GN07	0.00%	0.00%	0.00%	0.00%	0.00%	0.00%	0.00%	0.00%	0.00%	0.00%	0.00%	0.00%	0.00%	0.00%	0.00%	0.00%	0.00%	0.00%
k__Bacteria;p__GN02;c__IIB17	0.00%	0.00%	0.00%	0.00%	0.00%	0.00%	0.00%	0.00%	0.00%	0.00%	0.00%	0.00%	0.00%	0.00%	0.00%	0.00%	0.00%	0.00%
k__Bacteria;p__GN04;c__	0.00%	0.00%	0.00%	0.00%	0.00%	0.00%	0.00%	0.00%	0.00%	0.00%	0.00%	0.00%	0.00%	0.00%	0.00%	0.00%	0.00%	0.00%
k__Bacteria;p__GN04;c__GN15	0.00%	0.00%	0.00%	0.10%	0.10%	0.00%	0.00%	0.00%	0.00%	0.00%	0.00%	0.00%	0.00%	0.00%	0.00%	0.00%	0.00%	0.00%
k__Bacteria;p__GN04;c__MSB-5A5	0.00%	0.00%	0.00%	0.00%	0.00%	0.00%	0.00%	0.00%	0.00%	0.00%	0.00%	0.00%	0.00%	0.00%	0.00%	0.00%	0.00%	0.00%
k__Bacteria;p__GOUTA4;c__	0.00%	0.00%	0.00%	0.00%	0.00%	0.00%	0.00%	0.00%	0.00%	0.00%	0.00%	0.00%	0.00%	0.10%	0.00%	0.00%	0.00%	0.10%
k__Bacteria;p__Gemmatimonadetes;c__Gemm-2	0.00%	0.00%	0.00%	0.00%	0.00%	0.00%	0.00%	0.00%	0.00%	0.00%	0.00%	0.00%	0.00%	0.00%	0.00%	0.20%	0.20%	0.00%
k__Bacteria;p__Gemmatimonadetes;c__Gemm-3	0.00%	0.00%	0.00%	0.00%	0.00%	0.00%	0.00%	0.00%	0.00%	0.10%	0.10%	0.00%	0.00%	0.00%	0.00%	0.30%	0.10%	0.00%
k__Bacteria;p__Gemmatimonadetes;c__JL-ETNP-Z39	0.00%	0.00%	0.00%	0.00%	0.00%	0.00%	0.00%	0.00%	0.00%	0.00%	0.00%	0.00%	0.00%	0.00%	0.00%	0.00%	0.00%	0.00%
k__Bacteria;p__Kazan-3B-28;c__	0.00%	0.10%	0.00%	0.10%	0.00%	0.00%	0.00%	0.00%	0.00%	0.00%	0.00%	0.00%	0.00%	0.00%	0.00%	0.00%	0.00%	0.00%
k__Bacteria;p__LD1;c__	0.00%	0.00%	0.00%	0.00%	0.00%	0.00%	0.00%	0.00%	0.00%	0.00%	0.00%	0.00%	0.00%	0.00%	0.00%	0.00%	0.00%	0.00%
k__Bacteria;p__Lentisphaerae;c__[Lentisphaeria]	0.00%	0.00%	0.00%	0.00%	0.00%	0.00%	0.00%	0.00%	0.00%	0.00%	0.00%	0.00%	0.00%	0.00%	0.00%	0.00%	0.00%	0.00%
k__Bacteria;p__MVP-21;c__	0.00%	0.00%	0.00%	0.00%	0.00%	0.00%	0.00%	0.00%	0.00%	0.00%	0.00%	0.00%	0.00%	0.00%	0.00%	0.10%	0.00%	0.00%
k__Bacteria;p__MVS-104;c__	0.00%	0.00%	0.00%	0.00%	0.00%	0.00%	0.00%	0.00%	0.00%	0.00%	0.00%	0.00%	0.00%	0.00%	0.00%	0.00%	0.00%	0.00%
k__Bacteria;p__NC10;c__12-24	0.00%	0.00%	0.00%	0.10%	0.00%	0.00%	0.10%	0.00%	0.00%	0.00%	0.10%	0.00%	0.00%	0.10%	0.00%	0.00%	0.00%	0.00%
k__Bacteria;p__NKB19;Other	0.00%	0.00%	0.00%	0.00%	0.00%	0.00%	0.00%	0.00%	0.00%	0.00%	0.00%	0.00%	0.00%	0.00%	0.00%	0.00%	0.00%	0.00%
k__Bacteria;p__NKB19;c__	0.00%	0.00%	0.00%	0.10%	0.00%	0.00%	0.00%	0.00%	0.00%	0.00%	0.00%	0.00%	0.00%	0.00%	0.00%	0.00%	0.00%	0.00%
k__Bacteria;p__NKB19;c__TSBW08	0.00%	0.00%	0.00%	0.00%	0.00%	0.00%	0.00%	0.00%	0.00%	0.00%	0.00%	0.00%	0.00%	0.00%	0.00%	0.00%	0.00%	0.00%
k__Bacteria;p__OD1;c__Mb-NB09	0.00%	0.00%	0.00%	0.10%	0.00%	0.00%	0.00%	0.00%	0.00%	0.00%	0.00%	0.00%	0.00%	0.00%	0.00%	0.00%	0.00%	0.00%
k__Bacteria;p__OP11;c__	0.00%	0.00%	0.00%	0.00%	0.00%	0.00%	0.00%	0.00%	0.00%	0.00%	0.00%	0.00%	0.00%	0.00%	0.00%	0.00%	0.00%	0.00%
k__Bacteria;p__OP11;c__OP11-1	0.00%	0.00%	0.00%	0.00%	0.00%	0.00%	0.00%	0.00%	0.00%	0.00%	0.00%	0.00%	0.00%	0.00%	0.00%	0.00%	0.00%	0.00%
k__Bacteria;p__OP11;c__OP11-2	0.00%	0.00%	0.00%	0.00%	0.00%	0.00%	0.00%	0.00%	0.00%	0.00%	0.00%	0.00%	0.00%	0.00%	0.00%	0.00%	0.00%	0.00%
k__Bacteria;p__OP11;c__OP11-3	0.00%	0.00%	0.00%	0.00%	0.00%	0.00%	0.00%	0.00%	0.00%	0.00%	0.00%	0.00%	0.00%	0.00%	0.00%	0.00%	0.00%	0.00%
k__Bacteria;p__OP11;c__OP11-4	0.00%	0.00%	0.00%	0.00%	0.00%	0.00%	0.00%	0.00%	0.00%	0.00%	0.00%	0.00%	0.00%	0.00%	0.00%	0.00%	0.00%	0.00%
k__Bacteria;p__OP11;c__WCHB1-64	0.00%	0.00%	0.00%	0.00%	0.00%	0.00%	0.00%	0.00%	0.00%	0.00%	0.00%	0.00%	0.00%	0.00%	0.00%	0.00%	0.00%	0.00%
k__Bacteria;p__OP3;c__BD4-9	0.00%	0.00%	0.00%	0.00%	0.10%	0.00%	0.00%	0.00%	0.00%	0.00%	0.00%	0.00%	0.00%	0.00%	0.00%	0.00%	0.00%	0.00%
k__Bacteria;p__OP3;c__PBS-25	0.00%	0.00%	0.00%	0.00%	0.00%	0.00%	0.00%	0.00%	0.00%	0.00%	0.00%	0.10%	0.00%	0.00%	0.00%	0.00%	0.00%	0.00%
k__Bacteria;p__OP8;c__OP8_1	0.00%	0.00%	0.00%	0.00%	0.00%	0.00%	0.00%	0.00%	0.00%	0.00%	0.00%	0.00%	0.00%	0.00%	0.00%	0.00%	0.00%	0.00%
k__Bacteria;p__OP8;c__OP8_2	0.00%	0.00%	0.00%	0.00%	0.00%	0.00%	0.00%	0.00%	0.00%	0.00%	0.00%	0.00%	0.00%	0.00%	0.00%	0.00%	0.00%	0.00%
k__Bacteria;p__Planctomycetes;c__	0.00%	0.00%	0.00%	0.00%	0.00%	0.00%	0.00%	0.00%	0.00%	0.00%	0.00%	0.00%	0.00%	0.00%	0.00%	0.00%	0.00%	0.00%
k__Bacteria;p__Planctomycetes;c__028H05-P-BN-P5	0.00%	0.00%	0.00%	0.00%	0.00%	0.00%	0.00%	0.00%	0.00%	0.00%	0.00%	0.00%	0.00%	0.00%	0.00%	0.00%	0.00%	0.00%
k__Bacteria;p__Planctomycetes;c__BD7-11	0.00%	0.00%	0.00%	0.00%	0.00%	0.00%	0.00%	0.00%	0.00%	0.10%	0.00%	0.10%	0.10%	0.10%	0.00%	0.00%	0.00%	0.00%
k__Bacteria;p__Planctomycetes;c__C6	0.00%	0.00%	0.00%	0.10%	0.10%	0.00%	0.00%	0.00%	0.00%	0.00%	0.00%	0.10%	0.00%	0.00%	0.00%	0.00%	0.00%	0.00%
k__Bacteria;p__Planctomycetes;c__ODP123	0.00%	0.00%	0.00%	0.10%	0.10%	0.00%	0.00%	0.00%	0.00%	0.00%	0.00%	0.00%	0.00%	0.00%	0.00%	0.00%	0.00%	0.00%
k__Bacteria;p__Planctomycetes;c__OM190	0.00%	0.00%	0.10%	0.10%	0.10%	0.00%	0.00%	0.00%	0.00%	0.00%	0.00%	0.10%	0.10%	0.00%	0.00%	0.10%	0.10%	0.00%
k__Bacteria;p__Planctomycetes;c__Pla3	0.00%	0.00%	0.00%	0.00%	0.00%	0.00%	0.00%	0.00%	0.00%	0.00%	0.00%	0.00%	0.00%	0.00%	0.00%	0.00%	0.00%	0.00%
k__Bacteria;p__Planctomycetes;c__Pla4	0.00%	0.00%	0.00%	0.00%	0.00%	0.00%	0.00%	0.00%	0.00%	0.00%	0.00%	0.00%	0.00%	0.00%	0.00%	0.00%	0.00%	0.00%
k__Bacteria;p__Planctomycetes;c__vadinHA49	0.00%	0.10%	0.00%	0.10%	0.00%	0.00%	0.00%	0.00%	0.00%	0.20%	0.00%	0.00%	0.10%	0.00%	0.00%	0.00%	0.00%	0.00%
k__Bacteria;p__Proteobacteria;Other	0.00%	0.00%	0.00%	0.00%	0.00%	0.00%	0.00%	0.00%	0.00%	0.00%	0.00%	0.00%	0.00%	0.00%	0.00%	0.00%	0.00%	0.00%
k__Bacteria;p__Proteobacteria;c__	0.00%	0.10%	0.00%	0.00%	0.00%	0.00%	0.00%	0.00%	0.00%	0.00%	0.00%	0.10%	0.00%	0.00%	0.00%	0.00%	0.00%	0.00%
k__Bacteria;p__Proteobacteria;c__TA18	0.00%	0.00%	0.00%	0.00%	0.00%	0.00%	0.00%	0.00%	0.00%	0.00%	0.10%	0.10%	0.00%	0.10%	0.10%	0.00%	0.00%	0.10%
k__Bacteria;p__Proteobacteria;c__Zetaproteobacteria	0.00%	0.00%	0.00%	0.00%	0.00%	0.00%	0.00%	0.00%	0.00%	0.00%	0.00%	0.00%	0.00%	0.00%	0.00%	0.00%	0.00%	0.00%
k__Bacteria;p__SBR1093;c__	0.00%	0.00%	0.00%	0.00%	0.00%	0.00%	0.00%	0.00%	0.00%	0.00%	0.00%	0.00%	0.00%	0.00%	0.00%	0.00%	0.00%	0.00%
k__Bacteria;p__SBR1093;c__VHS-B5-50	0.00%	0.00%	0.10%	0.00%	0.00%	0.00%	0.00%	0.00%	0.00%	0.00%	0.00%	0.00%	0.00%	0.00%	0.00%	0.00%	0.00%	0.00%
k__Bacteria;p__SC4;c__	0.00%	0.00%	0.00%	0.10%	0.10%	0.00%	0.00%	0.00%	0.00%	0.00%	0.10%	0.00%	0.00%	0.10%	0.10%	0.00%	0.00%	0.10%
k__Bacteria;p__SR1;c__	0.00%	0.00%	0.00%	0.00%	0.00%	0.00%	0.00%	0.00%	0.00%	0.00%	0.00%	0.00%	0.00%	0.00%	0.00%	0.00%	0.00%	0.00%
k__Bacteria;p__Spirochaetes;c__GN05	0.00%	0.00%	0.00%	0.00%	0.00%	0.00%	0.00%	0.00%	0.00%	0.00%	0.00%	0.00%	0.00%	0.00%	0.00%	0.00%	0.00%	0.00%
k__Bacteria;p__Spirochaetes;c__MVP-15	0.00%	0.00%	0.00%	0.00%	0.00%	0.00%	0.00%	0.00%	0.00%	0.00%	0.00%	0.00%	0.00%	0.00%	0.00%	0.00%	0.00%	0.00%
k__Bacteria;p__Spirochaetes;c__[Brevinematae]	0.00%	0.00%	0.00%	0.00%	0.00%	0.00%	0.00%	0.00%	0.00%	0.00%	0.00%	0.00%	0.00%	0.00%	0.00%	0.00%	0.00%	0.00%
k__Bacteria;p__Spirochaetes;c__[Leptospirae]	0.00%	0.00%	0.00%	0.20%	0.00%	0.00%	0.00%	0.00%	0.00%	0.00%	0.00%	0.00%	0.00%	0.00%	0.00%	0.00%	0.00%	0.00%
k__Bacteria;p__TM6;c__	0.00%	0.00%	0.00%	0.00%	0.00%	0.00%	0.00%	0.00%	0.00%	0.00%	0.00%	0.00%	0.00%	0.00%	0.00%	0.00%	0.00%	0.00%
k__Bacteria;p__TM6;c__SBRH58	0.00%	0.00%	0.00%	0.00%	0.00%	0.00%	0.00%	0.00%	0.00%	0.00%	0.00%	0.00%	0.00%	0.10%	0.00%	0.00%	0.00%	0.00%
k__Bacteria;p__TM7;Other	0.00%	0.00%	0.00%	0.00%	0.00%	0.00%	0.00%	0.00%	0.00%	0.00%	0.00%	0.00%	0.00%	0.00%	0.00%	0.00%	0.00%	0.00%
k__Bacteria;p__TM7;c__	0.00%	0.10%	0.00%	0.00%	0.00%	0.00%	0.00%	0.00%	0.00%	0.20%	0.00%	0.00%	0.10%	0.00%	0.00%	0.10%	0.00%	0.00%
k__Bacteria;p__TM7;c__MJK10	0.00%	0.00%	0.00%	0.00%	0.00%	0.00%	0.00%	0.00%	0.00%	0.00%	0.00%	0.00%	0.00%	0.00%	0.00%	0.00%	0.00%	0.00%
k__Bacteria;p__Tenericutes;c__Mollicutes	0.00%	0.00%	0.00%	0.00%	0.00%	0.00%	0.00%	0.00%	0.00%	0.10%	0.00%	0.00%	0.00%	0.00%	0.00%	0.00%	0.00%	0.00%
k__Bacteria;p__Verrucomicrobia;Other	0.00%	0.00%	0.00%	0.00%	0.00%	0.00%	0.00%	0.00%	0.00%	0.00%	0.00%	0.00%	0.00%	0.00%	0.00%	0.00%	0.00%	0.00%
k__Bacteria;p__Verrucomicrobia;c__	0.00%	0.00%	0.00%	0.00%	0.00%	0.00%	0.00%	0.00%	0.00%	0.00%	0.00%	0.00%	0.00%	0.00%	0.00%	0.00%	0.00%	0.00%
k__Bacteria;p__Verrucomicrobia;c__Verruco-5	0.00%	0.00%	0.00%	0.00%	0.00%	0.00%	0.00%	0.00%	0.00%	0.00%	0.00%	0.00%	0.00%	0.00%	0.00%	0.00%	0.00%	0.00%
k__Bacteria;p__Verrucomicrobia;c__[Methylacidiphilae]	0.00%	0.00%	0.10%	0.00%	0.00%	0.00%	0.00%	0.00%	0.00%	0.00%	0.10%	0.10%	0.10%	0.10%	0.00%	0.00%	0.00%	0.10%
k__Bacteria;p__WS2;c__	0.00%	0.00%	0.00%	0.00%	0.00%	0.00%	0.00%	0.00%	0.00%	0.00%	0.00%	0.00%	0.00%	0.00%	0.00%	0.00%	0.00%	0.00%
k__Bacteria;p__WS2;c__Kazan-3B-09	0.00%	0.00%	0.00%	0.00%	0.00%	0.00%	0.00%	0.00%	0.00%	0.00%	0.00%	0.00%	0.00%	0.00%	0.00%	0.00%	0.00%	0.00%
k__Bacteria;p__WS2;c__SHA-109	0.00%	0.00%	0.00%	0.10%	0.00%	0.00%	0.00%	0.00%	0.00%	0.10%	0.00%	0.00%	0.00%	0.00%	0.00%	0.00%	0.00%	0.00%
k__Bacteria;p__WS4;c__	0.00%	0.00%	0.00%	0.00%	0.00%	0.00%	0.00%	0.00%	0.00%	0.00%	0.00%	0.00%	0.00%	0.00%	0.00%	0.00%	0.00%	0.00%
k__Bacteria;p__WS5;c__	0.00%	0.00%	0.00%	0.00%	0.00%	0.00%	0.00%	0.00%	0.00%	0.00%	0.00%	0.00%	0.00%	0.00%	0.00%	0.00%	0.00%	0.00%
k__Bacteria;p__WS6;c__	0.00%	0.00%	0.00%	0.00%	0.00%	0.00%	0.00%	0.00%	0.00%	0.00%	0.00%	0.00%	0.00%	0.00%	0.00%	0.00%	0.00%	0.00%
k__Bacteria;p__WS6;c__B142	0.00%	0.00%	0.00%	0.00%	0.00%	0.00%	0.00%	0.00%	0.00%	0.00%	0.00%	0.00%	0.00%	0.00%	0.00%	0.00%	0.00%	0.00%
k__Bacteria;p__WS6;c__SC72	0.00%	0.00%	0.00%	0.00%	0.00%	0.00%	0.00%	0.00%	0.00%	0.00%	0.00%	0.00%	0.00%	0.00%	0.00%	0.00%	0.00%	0.00%
k__Bacteria;p__WWE1;c__[Cloacamonae]	0.00%	0.00%	0.00%	0.00%	0.00%	0.00%	0.00%	0.00%	0.00%	0.00%	0.00%	0.00%	0.00%	0.00%	0.00%	0.00%	0.00%	0.00%
k__Bacteria;p__ZB3;c__	0.00%	0.00%	0.00%	0.00%	0.00%	0.00%	0.00%	0.00%	0.00%	0.00%	0.00%	0.00%	0.00%	0.00%	0.00%	0.00%	0.00%	0.00%
k__Bacteria;p__ZB3;c__BS119	0.00%	0.00%	0.00%	0.00%	0.00%	0.00%	0.00%	0.00%	0.00%	0.00%	0.00%	0.00%	0.00%	0.00%	0.00%	0.00%	0.00%	0.00%
k__Bacteria;p__[Caldithrix];c__KSB1	0.00%	0.00%	0.00%	0.00%	0.00%	0.00%	0.00%	0.00%	0.00%	0.00%	0.00%	0.00%	0.00%	0.00%	0.00%	0.00%	0.00%	0.00%
k__Bacteria;p__[Thermi];c__Deinococci	0.00%	0.00%	0.00%	0.00%	0.00%	0.00%	0.00%	0.00%	0.00%	0.00%	0.00%	0.00%	0.00%	0.00%	0.00%	0.00%	0.00%	0.00%
